# *Asparagus officinalis* L. extract exhibits anti-proliferative and anti-invasive effects in endometrial cancer cells and a transgenic mouse model of endometrial cancer

**DOI:** 10.3389/fphar.2024.1507042

**Published:** 2024-12-04

**Authors:** Ziwei Fang, Weimin Kong, Ziyi Zhao, Wenchuan Sun, Guangxu Xu, Leslie H. Clark, Stephanie A. Sullivan, Arthur-Quan Tran, Chang-Sheng Zhou, Delin Sun, Luyu Zhao, Jiandong Wang, Chunxiao Zhou, Victoria L. Bae-Jump

**Affiliations:** ^1^ Department of Gynecology, Beijing Obstetrics and Gynecology Hospital, Capital Medical University, Beijing Maternal and Child Healthcare Hospital, Beijing, China; ^2^ Division of Gynecologic Oncology, University of North Carolina at Chapel Hill, Chapel Hill, NC, United States; ^3^ Department of Gynecology, Shanghai University of Medicine and Health Sciences Affiliated Sixth People’s Hospital South Campus, Shanghai, China; ^4^ Shandong Juxinyuan Asparagus Industry Development Research Institute, HeZe, Shandong, China; ^5^ Shandong Juxinyuan Agricultural Technology Co, LTD., HeZe, Shandong, China; ^6^ Lineberger Comprehensive Cancer Center, University of North Carolina at Chapel Hill, Chapel Hill, NC, United States

**Keywords:** *Asparagus officinalis*, endometrial cancer, apoptosis, invasion, synergy, tumor growth

## Abstract

**Introduction:**

Endometrial cancer is the most common malignancy of the female reproductive system in the United States. *Asparagus officinalis* is a versatile, nutrient-dense, low-calorie vegetable that contains various bioactive metabolites that have shown a variety of biologic functions beneficial to health. The metabolites from *asparagus officinalis* extracts or *asparagus officinalis* extracts exhibit significant anti-tumorigenic activity in some pre-clinical models of cancer.

**Methods:**

Endometrial cancer cells were used to study the effects of *asparagus officinalis* on anti-proliferation, anti-invasion and increased sensitivity to cisplatin, and obese and lean *Lkb1* ^*fl/fl*^ *p53* ^*fl/fl*^ mouse model of endometrial cancer was used to study the role of asparagus officinalis in tumor growth.

**Results:**

Treatment with increasing concentrations of *Asparagus officinalis* extracts significantly inhibited cell proliferation, reduced glycolytic activity, induced cellular stress and apoptosis, caused cell cycle G1 arrest, increased the sensitivity of cells to cisplatin, reduced cell adhesion and invasion, and activation of AMPK and inhibition of the AKT/mTOR and MAPK signaling pathways in endometrial cancer cells. Moreover, *asparagus officinalis* extracts suppressed cell adhesion and invasion through the modulation of the epithelial-to-mesenchymal transition process. *Asparagus officinalis* extract treatment for 4 weeks resulted in a significant reduction in tumor growth in *Lkb1* ^*fl/fl*^ *p53* ^*fl/fl*^ mice under both obese and lean conditions, with a decrease in Ki-67 and vascular endothelial growth factor expression and an increase in Bip expression in endometrial tumors.

**Discussion:**

These findings provide strong preclinical evidence for the potential therapeutic benefit of *asparagus officinalis* extract as a novel dietary strategy in the treatment of endometrial cancer. Further clinical trials of dietary intervention of *asparagus officinalis* or combination with cisplatin in patients with endometrial cancer are warranted.

## Introduction

Endometrial cancer (EC) is the most common gynecologic malignancy among women in the United States, with an estimated 67,880 new cases and 13,250 deaths in the United States in 2024. The morbidity and mortality of EC are strongly associated with obesity and are on the rise, due to the alarmingly high rates of obesity in the US population (Siegel et al., 2024). Most endometrial cancers are diagnosed early and cured with surgery (with or without radiation therapy). The five-year survival rate of early-stage patients is greater than 90%, while the prognosis of advanced and recurrent endometrial cancer is poor, with a five-year survival rate as low as 20%, making EC the sixth leading cause of cancer death in women in the US (Tronconi et al., 2022). Patients with advanced and recurrent EC respond poorly to cytotoxic chemotherapy, resulting in limited effective treatment options available for these patients; and thus, the development of alternative therapeutic approaches will help improve the survival of patients with advanced and recurrent EC.

Plants are the most widely used natural resources in the development of new anti-tumor agents due to their availability and abundance. Moreover, plants have been considered safer than synthetic compounds in clinical practices. At least one-half of all anti-cancer drugs on the market come from natural sources (Sharifi-Rad et al., 2019). *Asparagus officinalis* L. (ASP) is a popular plant widely grown as a heathy and nutritious vegetable that is rich in metabolites, including flavonoids, polysaccharides, saponins, and other phenolics. Studies on ASP extracts have shown that it has a variety of beneficial biological activities, such as antioxidant, immunomodulatory, hypoglycemic, hypolipidemic, antihypertensive, antiepileptic, antibacterial, antiviral and anti-inflammatory effects (Guo et al., 2020; Hamdi et al., 2021). An epidemiologic study confirmed that long-term consumption of vegetables, including ASP, was inversely associated with the risk of liver cancer in the Chinese population (Zhang et al., 2013). Among the herbal extracts of grape, grapefruit, rue, black seed, rhubarb, green tea, mustard seed, arugula, and avocado pulp, ASP extract was the most effective plant for inhibiting cell proliferation and increasing the sensitivity of cells to doxorubicin in liver cancer HepG2 cells (Al-Shafie et al., 2023). Importantly, there is recent increasing evidence that ASP extracts or metabolites derived from ASP exhibit significant antitumor activity in various types of preclinical cancer models. Our previous studies showed that ASP decreased cellular viability, caused cell cycle G1 phase arrest, induced apoptosis, reduced cell invasion, and inhibited tumor growth in ovarian cancer cells and in a transgenic mouse model of ovarian cancer. Meanwhile, ASP combined with paclitaxel synergistically inhibited cell proliferation, induced cellular stress and apoptosis, and reduced cell invasion in paclitaxel-sensitive and -resistant ovarian cancer cell lines (Xu et al., 2021; Zhang et al., 2023).

Given that obesity and its associated metabolic abnormalities are hallmarks of EC, and ASP has the potential to improve abnormal metabolism in cancer cells, we speculate that if ASP has antitumor activity in EC and improves glycolysis in EC cells, EC patients, especially obese patients, will benefit greatly (Al-Shafie et al., 2023; King et al., 2023). Thus, in this study, we investigated the effects of ASP on cell proliferation, apoptosis, cellular stress, invasion, and glucose metabolism in EC cell lines and in a transgenic mouse model of EC under obese and lean conditions.

## Materials and methods

### Cell culture and chemicals

EC cell lines KLE, ECC-1 and HEC-1A were originally obtained from the American Type Culture Collection (ATCC), and the Ishikawa cells were purchased from Sigma-Aldrich (St. Louis, MO). The KLE cells were cultured in DMEM/F12 medium with 10% fetal bovine serum (FBS). The HEC-1A cells were maintained in McCoy’s 5A medium with 10% FBS. The ECC-1 cells were cultured in 1,640 medium with 5% FBS. The Ishikawa cells were maintained in MEM with 10% FBS at 37°C under 5% CO_2_. All cell lines are authenticated annually by LabCorp (Burlington, NC) using short tandem repeat (STR) profiling, and *mycoplasma* tests were performed in these cell lines every 6 months. All antibodies used for *in vitro* studies were purchased from Cell Signaling Technology (Danvers, MA). All chemicals were from Thermo Fisher Scientific (Waltham, MA).

### Preparation of ASP extract

The ASP extract was obtained from Shandong Juxinyuan Asparagus Industry Development Research Institute, P.R. China. Asparagus stems and shoots were used to prepare ASP extracts following the protocol of our previous publication (Xu et al., 2021). Briefly, the crushed stems and shoots of ASP were rinsed in distilled water at 95°C before being extracted using squeezer equipment. After centrifuging to remove insoluble solids, the crude extract was concentrated in vacuum to produce a concentrated solution containing 20% soluble solids. This concentrated extract is further concentrated through a water bath. Finally, the concentrated extract includes 70% soluble components and is sterilized through pasteurization (Supplementary Table S1). After the ASP extraction was completed, the sample of ASP was sent to local government agencies to examine 11 pesticide components. There was no pesticide ingredients found in the ASP extract used in this study (Xu et al., 2021).

### HPLC analysis

The HPLC analysis system (Shimadzu LC 20AT, Columbia, MD) equipped with a vacuum degasser, a quaternary pump, an autosampler, and a diode array detector or ELSD (for flavonoids) was used to qualitatively detect flavonoids, saponins, phenolic compounds and other active metabolites in the ASP extracts. All samples were filtered through 0.45 µm PTFE filters before HPLC analysis. Chromatographic separations were performed on an Agilent ZORBAXSB-C18 column (4.6 × 250 mm, 5 µm). The mobile phase was a mixture of methanol and water (10%) or a mixture of formic acid and water (2%, for flavonoids). The temperature of the column was 25°C. The detection wavelengths range were from 210–500 nm. The flow rate was 1 mL/min. All measurements were performed in triplicate. Data acquisition was performed using the Shimadzu LCsolution software program. All measurements of ASP extracts were performed in Institute of Oceanology, Chinese Academy of Science (Qingdao, China, Supplementary Table S2). The major bioactive metabolites were shown in Table 1.

**TABLE 1:** Major bioactive metabolites.

Bioactive metabolites	ASP extracts
Rutin	6,370^a^
Quercetin	136^a^
Sarsagenin	1773^a^
Resveratrol	130.8^a^
Saponins	198.9^b^
Polysaccharides	2,590.4^b^
Phenolic compounds	605^b^
Free amino acids	1,430^b^
Molybdenum	40^a^
selenium	0.1526^a^

^a^ ug/100 mL.

^b^ mg/100 mL.

### MTT assay

The KLE, ECC-1 and HEC-1A and Ishikawa Cells were seeded in 96-well plates, with 4,000–6,000 cells per well for 24 h at 37°C. The cells were then treated with the indicated doses (0.001, 0.01.0.1, 0.5, 0.75.1, 5 and 10 mg/mL) of ASP for 72 h. Each treatment was tested in quadruplicate replicates. 5 μL MTT (5 mg/mL) was added to each well for 1 hour at 37°C. After incubation, the formazan crystals are dissolved in 100 µL dimethyl sulfoxide (DMSO)/well. Absorbance at 575 nm on 96-well plates was measured using a microplate reader (Tecan, Durham, NC). The IC_50_ value for the KLE, ECC-1 and HEC-1A and Ishikawa cells was calculated using the IC_50_ Calculator (AAT Bioquest, Sunnyvale, CA). Bliss independence model was used to evaluate the synergistic effect of ASP combined with rapamycin on cell proliferation (Foucquier and Guedj, 2015). CI = 1 (additive), CI < 1 (synergistic), or CI > 1 (antagonistic).

### Cell cycle assay

The KLE and Ishikawa cells were treated with 0.01, 0.1, and 1 mg/mL ASP extracts for 36 h in 5% CO_2_ at 37°C. The cells were harvested with 0.5% trypsin, fixed with 90% ice cold methanol for 1 hour, and then incubated in a Propidium Iodide/RNAse staining solution (Nexcelom Bioscience, Lawrence, MA) for 20 min. Cell cycles were detected by Cellometer (Nexcelom Bioscience). The data were analyzed by FCS4 Express (*De Novo* Software, Pasadena, CA).

## Annexin V assay

Analysis of Annexin-V expression in ASP-treated or untreated cells was performed using Cellometer. The KLE and Ishikawa cells were treated with 0.01, 0.1, and 0.5 mg/mL ASP extracts for 16 h. The cells were harvested with 0.25% trypsin and centrifuged at 1,200 rpm for 5 minutes. The cell pellet was resuspended in 100 µL of Annexin-V and propidium iodide (PI) double-stain solution for 15 min at room temperature. Analysis of apoptosis was performed by FCS4 Express software.

### Cleaved caspase 3, 8 and 9 assays

The KLE and Ishikawa cells were plated in 6-well plates at the concentration of 2.5 × 10^5^ cells/well overnight. The cells were exposed to 0.01,0.1 and 1 mg/mL ASP for 12 h and lysed with 1X caspase lysis buffer. BCA assay (Thermo Fisher) was used to detect protein concentrations. 150 μL of cell lysate was added to each well in a black 96-well plate, and reaction buffers with cleaved caspase 3, 8 and 9 substrates were mixed with the cell lysate at 37°C in the dark for 20 min. The fluorescence intensity for cleaved caspase 3 (Ex/Em = 400/505), cleaved caspase 8 (Ex/Em = 376/482), and cleaved caspase 9 (Ex/Em = 341/441) were recorded using a Tecan microplate reader.

### Reactive oxygen species assay

Intracellular reactive oxygen species (ROS) production was determined using the DCFH-DA assay. The KLE and Ishikawa cells were cultured in black 96-well plates overnight and treated with ASP extracts for 12 h 200 μM ROS inducer (pyocyanin) was used as a positive control. The cells were incubated with 20 µM DCFH-DA solution for 30 min. Cellular ROS generation was measured at an excitation wavelength of 485 nm and an emission wavelength of 530 nm using a Tecan plate reader. Cells were incubated with 20 µM DCFH-DA solution for 30 min.

### TMRE assay

Mitochondrial membrane potential was assayed using the TMRE assay (AAT Bioquest, Pleasanton, CA). The KLE and Ishikawa cells were seeded in a 96-well plate (10,000/well) overnight and incubated with 0.01, 0.1, and 1 mg/mL ASP extracts for 12 h. For positive control, we treated the cells with 20 µM FCCP for 30 min. After 100 µL of culture medium with 500 µM TMRE was added to each well and then incubated at 37°C for 30 min. TMRE fluorescence was measured using a Tecan plate reader at an excitation wavelength of 549 nm and an emission wavelength of 575 nm.

### Glucose uptake assay

The KLE and Ishikawa cells were plated into black 96-well plates at 6,000 cells/well overnight and treated with different concentrations of ASP extracts for 12 h. Each well on the 96-well plate was replaced with 100 µL glucose-free medium containing 100 ug/mL 2-NBDG for 20 min. The four wells without 2NBDG were used as negative controls. After washing each well with HBSS twice, fluorescence intensity (Ex/Em = 465/540) was measured via the Tecan plate reader.

### L-lactate assay

The content of L-lactate in culture medium in the KLE and Ishikawa cells was measured using the L-Lactate Assay Kit (Eton Bio, San Diego, CA) following the manufacturer’s instruction. In brief, the cells were treated with different concentrations of ASP extracts (0.01, 0.1, and 1 mg/mL) for 24 h. Subsequently, 25 µL of the sample medium was transferred into a 96-well plate and then 50 µL of distilled water and 25 µL of lactate assay solution were added to each well for 60 min at room temperature in the dark. The absorbance at 490 nm was measured using a Tecan microplate reader.

### ATP assay

Cellular ATP production was assessed using the Luminometric ATP Assay Kit (Fisher Scientific) following the manufacturer’s instruction. After the KLE and Ishikawa cells were treated with ASP extracts (0.01, 0.1, and 1 mg/mL) for 24 h in a 96-well plate, the ATP reaction mix was added to each well and incubated for 10–20 min in the dark. Cellular ATP production was measured with a Tecan plate reader. ATP levels were normalized based on the viable cell counts measured by the MTT assay.

### Adhesion assay

The KLE and Ishikawa cells were seeded in plates pre-coated with laminin-1 (Sigma-Aldrich) at 6,000 cells/well and treated with 0.01, 0.1, and 1 mg/mL ASP extracts for 2 h. After aspirating the supernatant, the cell lines were fixed with 5% glutaraldehyde for 30 min and then the plate was stained with 0.1% crystal violet solution for 15 min at room temperature. 100 μL of 10% acetic acid was added to each well to solubilize the dye. The plate was measured at a wavelength of 575 nm by a Tecan plate reader.

### Transwell assay

Transwell assay was conducted in 96-well plates pre-coated with 0.5-1 x BME (Trevigen, Gaithersburg, MD). The KLE and Ishikawa cells were starved in an FBS-free medium for 12 h and then plated in the upper chamber with a density of 3 × 10^4^. ASP extracts (0.01, 0.1, and 1 mg/mL) were added to the upper chambers and then the lower chambers were filled with regular medium. The plates were incubated for 6 h for cell invasion into the lower chambers at 37°C. 100 μL of calcein AM solution (Invitrogen, Carlsbad, CA) was added to the lower chambers and incubated for another 30 min. The plate was measured using a Tecan plate reader with an excitation/emission wavelength of 485/520 nm.

### Western blotting assay

After KLE and Ishikawa cells were treated with 0.01, 0.1, and 1 mg/mL ASP extracts for 24 h, the cells were lysed in RIPA buffer supplemented with protease inhibitors (Roche, Seattle, WA). Equal amounts of protein were separated by electrophoresis through 10%–12% sodium dodecyl sulfate–polyacrylamide gel electrophoresis gels and transferred into 0.2 µm PVDF membranes (Bio-Rad, Hercules, CA). After blocking with 5% non-fat milk at room temperature for 1 hour, the membranes were incubated with various primary antibodies overnight at 4°C. The primary antibodies were detected using horseradish peroxidase-conjugated secondary antibodies. Immunoblotting signals were visualized by Super Signal WestPico™ (Thermo Scientific) and analyzed using the Bio-Rad ChemiDoc™ image system.

### *Lkb1* ^*fl/fl*^ *p53* ^*fl/fl*^ mouse model of EC

The *Lkb1* ^*fl/fl*^ *p53* ^*fl/fl*^ mouse model of EC has been described previously (Guo et al., 2019). Our animal protocol (19–141) for this study was approved by the Institutional Animal Care and Use Committee at the University of North Carolina at Chapel Hill (UNC-CH). To induce endometrial tumors, 5 µL of 1 × 10^10^ pfu/mL Ad5-CMV-Cre (Transfer Vector Core, University of Iowa, IA) was injected into the left uterine horn of female *Lkb1* ^*fl/fl*^ *p53* ^*fl/fl*^ mice at 6–8 weeks of age. To compare the effect of concentrations of ASP extracts on tumor growth, the mice were initially divided into three groups (18 mice per group) and treated with 200 or 800 mg/kg ASP or vehicle orally (daily) for 4 weeks. Given that obesity is one of the leading risk factors for carcinogenesis and progression of EC, we explored the effect of ASP on tumor growth in *Lkb1* ^*fl/fl*^ *p53* ^*fl/fl*^ mice under obese and lean conditions. The mice were fed a high-fat diet (HFD) or a low-fat diet (LFD) starting at 3 weeks of age. Approximately 8 weeks after Ad5-CMV-Cre injection for tumor induction, mice were then divided into four groups (18 mice/group) and treated with 200 mg/kg ASP extract daily for 4 weeks: HFD control, HFD + ASP, LFD control, and LFD + ASP. Endometrial tumors and blood samples were collected and stored at −80°C until use. The control mice received the same volume of concentrated extract without ASP.

### Immunohistochemistry (IHC)

Bip, VEGF, phosphorylated-S6, and Ki67 antibodies were used for IHC staining on 4 mm paraffin sections of formalin-fixed tissue from the *LKB1* ^*fl/fl*^ *p53* ^*fl/fl*^ mice at the UNC-CH animal facility. All slides were scanned by Motic and analyzed by ImagePro software (Rockville, MD).

### Statistics

Data are presented as a mean ± the standard error of the mean. All experiments were repeated three times except for animal experiments. Graphs and statistical analysis were performed by GraphPad Prism 8 software. The unpaired Student’s t-test and analysis of variance (ANOVA) were performed for comparison between treatment and control groups in this study. *p* values < 0.05 were considered statistically significant.

## Results

### ASP inhibited cell proliferation and tumor growth in EC cells and the *Lkb1* ^*fl/fl*^ *p53* ^*fl/fl*^ mouse model of EC

**FIGURE 1:**
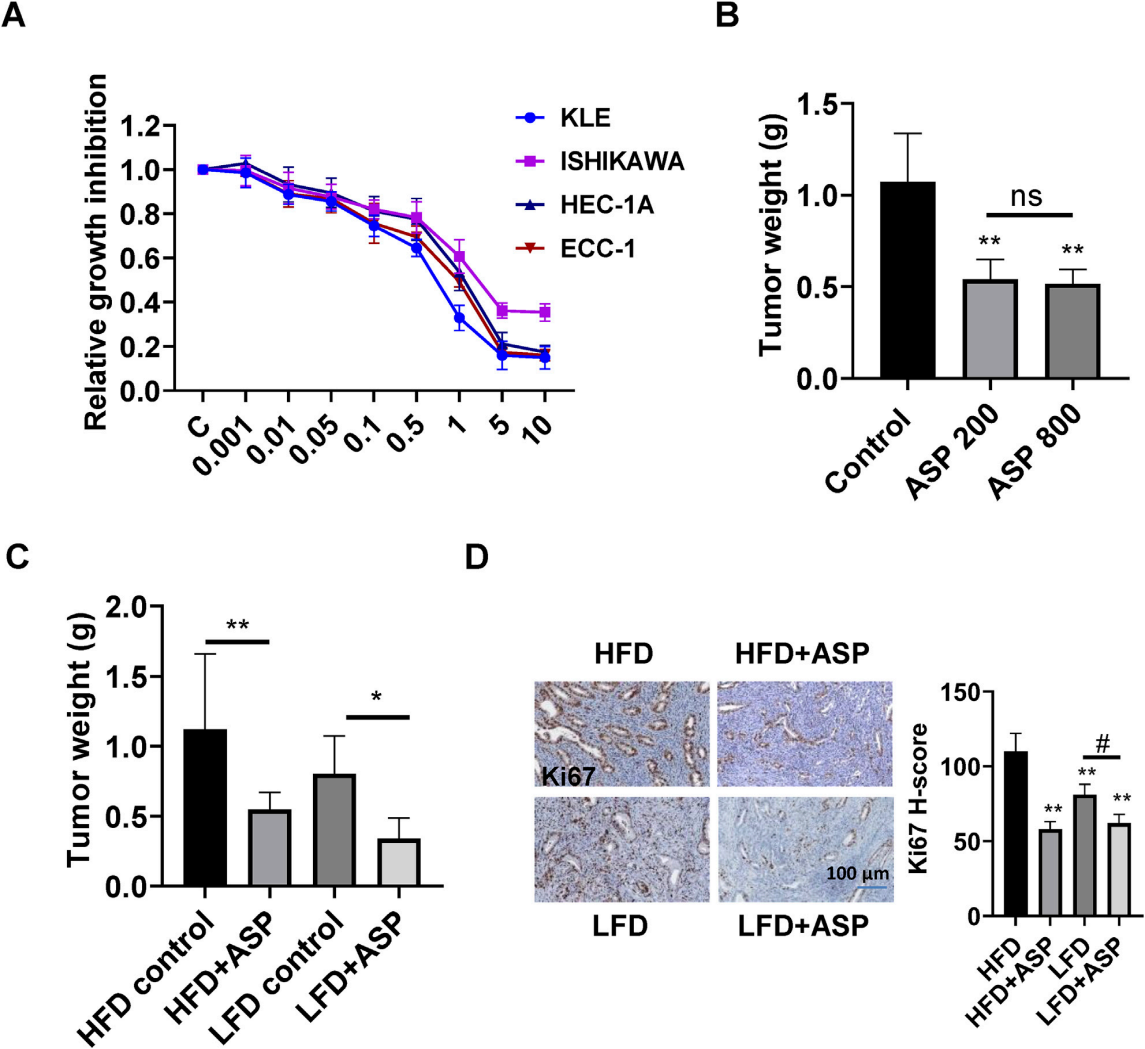
ASP extract inhibited cell proliferation and tumor growth in EC cells and *Lkb1* ^*fl/fl*^ *p53* ^*fl/fl*^ mice. MTT assay showed that ASP effectively reduced cell proliferation in a dose-dependent manner in KLE, Ishikawa, ECC-1, and HEC-1A cells after 72 h of treatment **(A)**. Daily treatment with ASP extract at a dose of 200 mg/kg or 800 mg/kg for 4 weeks significantly inhibited tumor growth in *Lkb1* ^*fl/fl*^ *p53* ^*fl/fl*^ mice **(B)**. ASP at a dose of 200 mg/kg for 4 weeks reduced tumor weight in obese and lean *Lkb1* ^*fl/fl*^ *p53* ^*fl/fl*^ mice **(C)**. ASP extract decreased Ki-67 expression in the endometrial tumors of obese and lean *Lkb1* ^*fl/fl*^ *p53* ^*fl/fl*^ mice **(D)**. *p < 0.05, **p < 0.01, ^#^ *p* < 0.05. * and ** represent comparison with control.

The anti-proliferative effects of different concentrations of ASP on the ECC-1, HEC-1A, KLE, and Ishikawa were evaluated using MTT assay. The results showed that ASP extracts significantly inhibited cell proliferation in a dose-dependent manner in all 4 cell lines after treatment with ASP for 72 h. The IC_50_ values of ASP in ECC-1, HEC-1A, KLE, and Ishikawa cells were 1.83, 2.91, 0.64, and 2.14 mg/mL, respectively (Figure 1A). To investigate whether ASP inhibits tumor growth *in vivo*, we treated *Lkb1* ^*fl/fl*^ *p53* ^*fl/fl*^ mice with 200 or 800 mg/kg ASP extract (oral gavage, daily) for 4 weeks. Compared with the vehicle group, the tumor weight of mice in the 200 mg/kg group was significantly reduced by 41.6% ± 6.2%, and the tumor weight of mice in the 800 mg/kg group was significantly reduced by 51.8% ± 7.1%. However, there was no statistical difference in tumor weight between the two treatment groups (Figure 1B). During ASP treatment, none of the mice showed abnormal activity, and no abnormalities were found in weekly body weight or blood glucose assessments (Supplementary Figure S1).

Given that obesity is an important contributor to carcinogenesis in EC and diet-induced obesity effectively promotes tumor growth in *Lkb1* ^*fl/fl*^ *p53* ^*fl/fl*^ mice (Guo et al., 2019; Pierce et al., 2021), the effect of ASP on tumor growth under obese and lean conditions was investigated in *Lkb1* ^*fl/fl*^ *p53* ^*fl/fl*^ mice. Before the treatment, the average body weight of the mice in the HFD was 35.5 g, and the average body weight in the LFD was 27.9 g. The obese and lean mice were treated with ASP extract (200 mg/kg, daily, oral gavage) for 4 weeks. Compared with the LFD group, the tumor weight in the HFD group increased significantly by 36.4%. In the HFD group, ASP effectively decreased tumor growth by 48.9%, while in the LFD group, ASP reduced tumor weight by 42.4% (Figure 1C). ASP exhibited greater anti-tumorigenic activity in obese mice compared with lean mice (*p* < 0.05). Similarly, further analysis of the expression of Ki-67 in EC tissues revealed that ASP was able to reduce the expression of Ki-67 by 47.2% in obese mice and 23.5% in lean mice (Figure 1D).

### ASP induced cell cycle G1 phase arrest in EC cells

**FIGURE 2:**
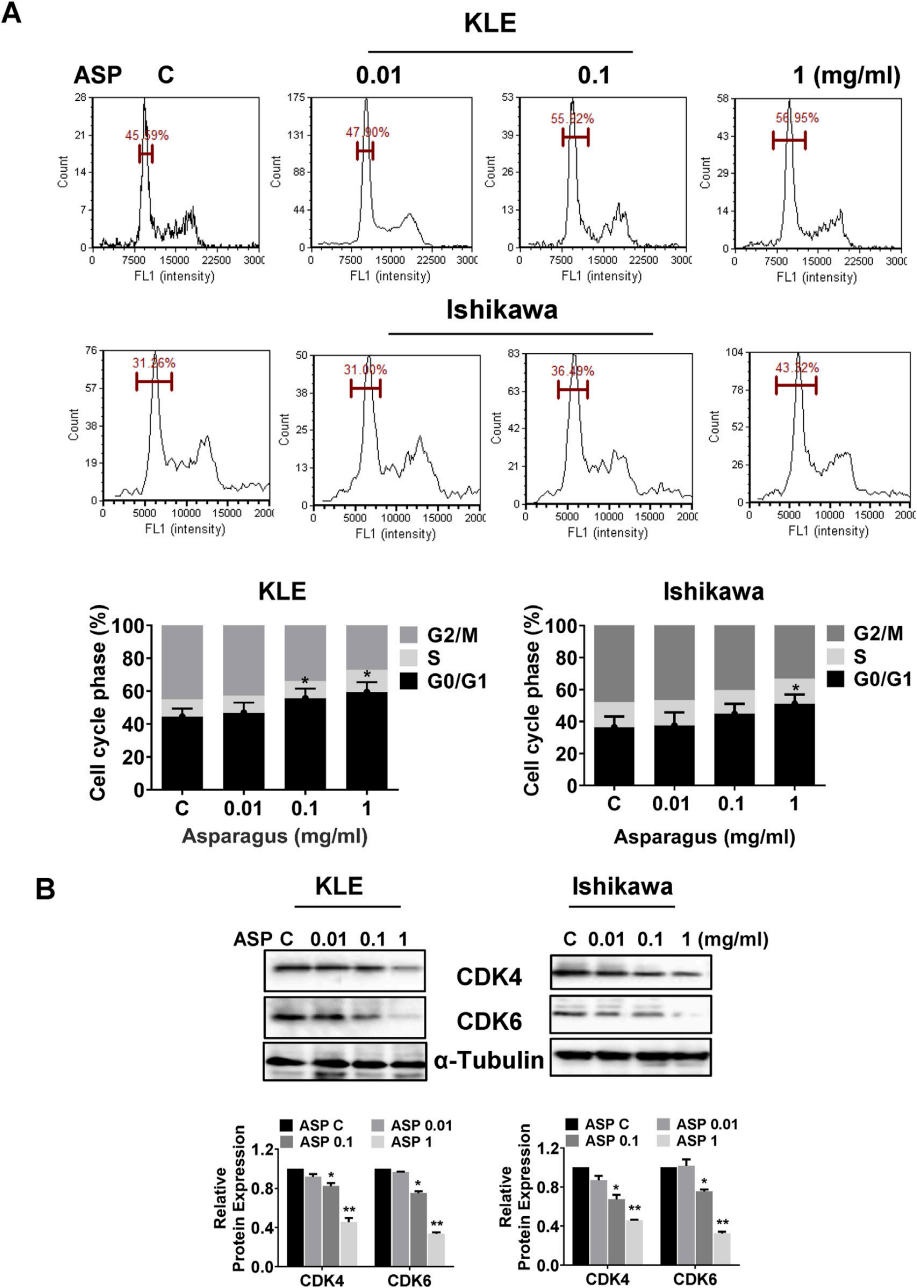
ASP caused cell cycle G1 arrest in EC cells. ASP extract caused cell cycle G1 arrest in the KLE and Ishikawa cells after 36 h of treatment **(A)**. Western blotting assay showed that ASP reduced the expression of CDK4 and CDK6 in both cell lines after 24 h of treatment **(B)**. *p < 0.05, **p < 0.01. * and ** represent comparison with control.

The KLE and Ishikawa were treated with 0.01, 0.1, and 1 mg/mL ASP extracts for 36 h, and an analysis of cell cycle prolife showed that ASP extract induced cell cycle G1 phase arrest in both cell lines (Figure 2A). Treatment of cells with 1 mg/mL ASP significantly increased G1 phase by 11.36% in the KLE cells and 12.06% in the Ishikawa cells, respectively. To understand the molecular mechanism underlying cell cycle G1 arrest induced by ASP, the expression of cell cycle-related proteins was detected by Western blotting assay after the KLE and Ishikawa cells were treated with ASP extract for 24 h. The results demonstrated that ASP extract reduced the expression of CDK4 and CDK6 in a concentration-dependent manner in both cell lines (Figure 2B). These results suggest that cell cycle G1 arrest is involved in ASP-induced inhibition of cell proliferation in EC cells.

### ASP induced apoptosis in EC cells

**FIGURE 3:**
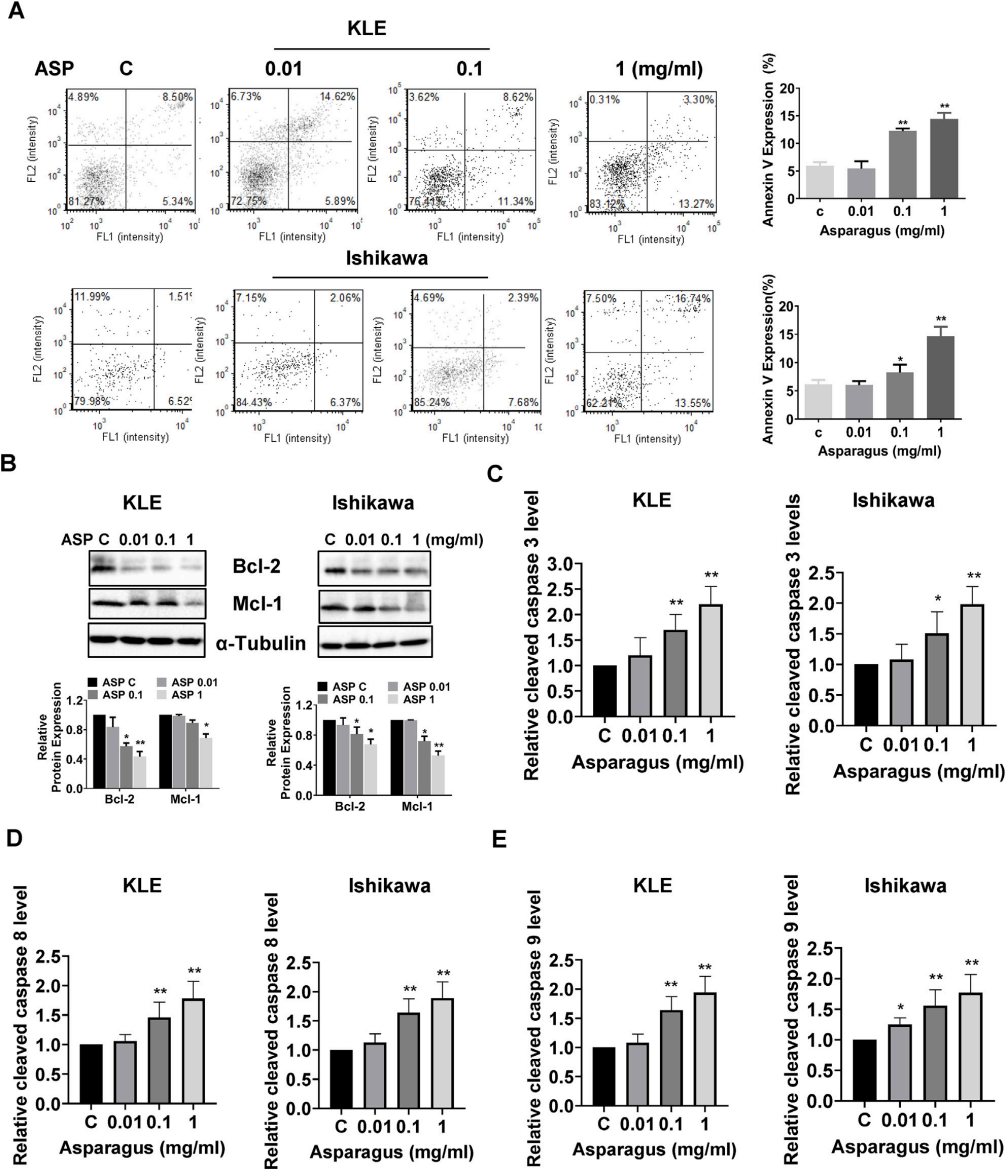
ASP induced apoptosis in EC cells. ASP extract increased the expression of Annexin V in the KLE and Ishikawa cells after 16 h of treatment **(A)**. Treatment of both cells with 0.01, 0.1, and 1 mg/mL ASP extract for 24 h resulted in decreased expression of Bcl-2 and Mcl-1 in both cell lines **(B)**. After treatment of KLE and Ishikawa cells with 0.01, 0.1, and 1 mg/mL for 12 h, ELISA assay demonstrated that ASP increased the activities of cleaved caspase 3, 8, and 9 in both cell lines **(C–E)**. *p < 0.05, **p < 0.01. * and ** represent comparison with control.

To investigate the effect of ASP on apoptosis, the KLE and Ishikawa cells were treated with varying concentrations of ASP for 16 h and then stained with Annexin V and PI. As shown in Figure 3A, a significant increase in early apoptotic cells was observed after treatment with 1 mg/mL ASP extract in both cell lines. These results were confirmed by Western blotting results. ASP extract reduced the expression of Bcl-2 and Mcl-1 in a concentration-dependent manner in both cell lines (Figure 3B). To examine whether ASP induced apoptosis through the extrinsic/intrinsic apoptotic pathway, the activities of cleaved caspase 3, 8, and 9 were assessed by ELISA assays. As shown in Figures 3C–E, the activities of cleaved caspase 3, 8, and 9 were increased in a dose-dependent manner by treatment with increasing concentrations of ASP for 12 h in both cell lines. 1 mg/mL ASP extract increased the activities of caspase 3, 8, and 9 by 120%, 78%, and 77% in the KLE cells and by 98%, 89%, and 94% in the Ishikawa cells, respectively. These results suggest that inhibition of cell proliferation by ASP depends on the induction of extrinsic and intrinsic apoptotic pathways in EC cells.

### ASP induced cellular stress in EC cells and *Lkb1* ^*fl/fl*^ *p53* ^*fl/fl*^ mice

**FIGURE 4:**
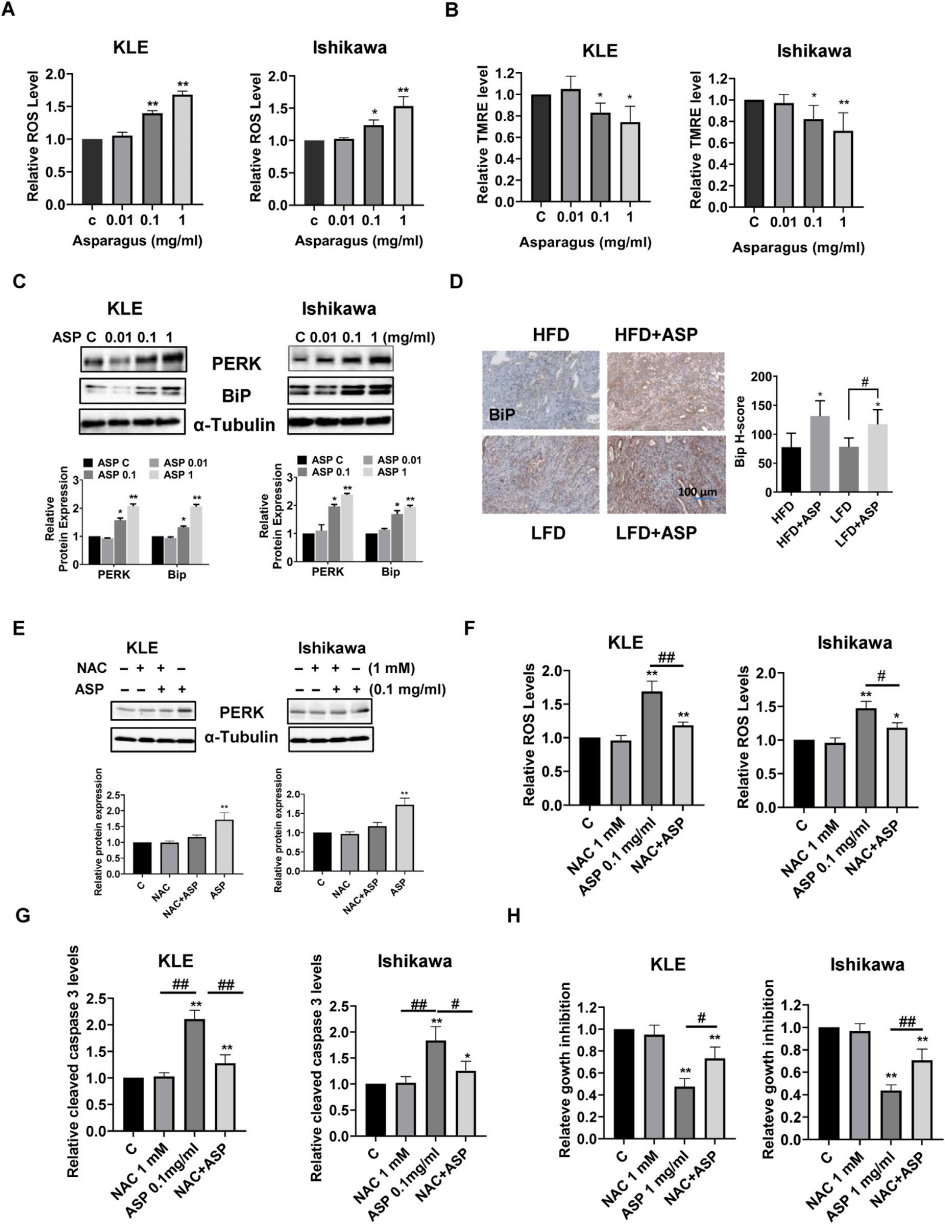
ASP induced cellular stress in EC. Treatment of ASP extract for 12 h effectively increased cellular ROS production in the KLE and Ishikawa cells **(A)**. The mitochondrial membrane potential was assessed by TMRE assay. Both cell lines were treated with ASP extracts for 12 h. TMRE assay showed that ASP significantly reduced mitochondrial membrane potential in both cell lines **(B)**. Western blotting results demonstrated that ASP increased the expression of BiP and PERK in both cell lines after 24 h of treatment **(C)**. IHC results showed that ASP increased BiP expression in EC tumor tissues of *LKB1* ^*fl/fl*^ *p53* ^*fl/fl*^ mice **(D)**. Pretreated with NAC for 4 h revised the ASP induced Bip expression **(E)**, ROS production **(F)**, and cleaved caspase 3 activity **(G)** in both cells. MTT assay showed that pretreated both cells with NAC partially revised ASP induced inhibition of cell proliferation **(H)** *p < 0.05, **p < 0.01. * and ** represent comparison with control. ^#^: *p* < 0.05, ^##^: *p* < 0.01 compared with each group.

Intercellular ROS production was measured after treatment of the KLE and Ishikawa cells with ASP extract for 12 h. ASP-treated cells showed a significant increase in ROS production in a concentration-dependent manner in both cell lines (Figure 4A). TMRE assay also showed that ASP extract reduced the mitochondrial membrane potential in both cell lines after 12 h of treatment. 1 mg/mL ASP effectively reduced mitochondrial membrane potential by 26% in the KLE cells and 29% in the Ishikawa cells compared to control cells, respectively (Figure 4B). Furthermore, after treatment of both cells with ASP for 24 h, Western blotting results showed that ASP extract increased the expression of cellular stress-related proteins PERK and Bip (Figure 4C). IHC results showed that there was no difference in Bip expression between obese and lean mice, but 4 weeks of ASP treatment increased Bip expression in both obese and lean mice (Figure 4D), indicating that ASP treatment significantly increased cellular stress in endometrial tumors.

Since ASP induced oxidant stress *in vitro and in vivo*, N-Acetylcysteine (NAC) was used to block ASP-induced cellular stress pathway to observe whether blocking the cellular stress pathway would reverse the inhibitory function of ASP on cell growth. The KLE and Ishikawa cells were pretreated with NAC (1 mM) for 4 h and then treated with ASP (0.1 mg/mL) for 12 h, Western blotting results showed that NAC significantly revised the ASP-induced PERK expression in both cells compared with control, NAC-treated, and ASP-treated groups (Figure 4E). The ROS assay confirmed that NAC partially reversed increased ROS production induced by 1 mg/mL ASP (Figure 4F). Similar results were found in cleaved caspase 3 assay. Pretreated with NAC effectively reduced cleaved caspase 3 activity by 82.1% in the KLE cells and 58% in the Ishikawa cells, respectively (Figure 4G). Importantly, the combination of NAC and ASP treatment partially reversed the inhibitory effect of 1 mg/mL ASP on cell proliferation, from 52.5% to 26.8% in the KLE cells, and 56.3%–29.6% in the Ishikawa cells, respectively (Figure 4H). These results suggest that cellular stress is associated with ASP-induced inhibition of cell proliferation and tumor growth in EC.

### ASP reduced cellular adhesion and invasion in EC cells and *Lkb1* ^*fl/fl*^ *p53* ^*fl/fl*^ mice

**FIGURE 5:**
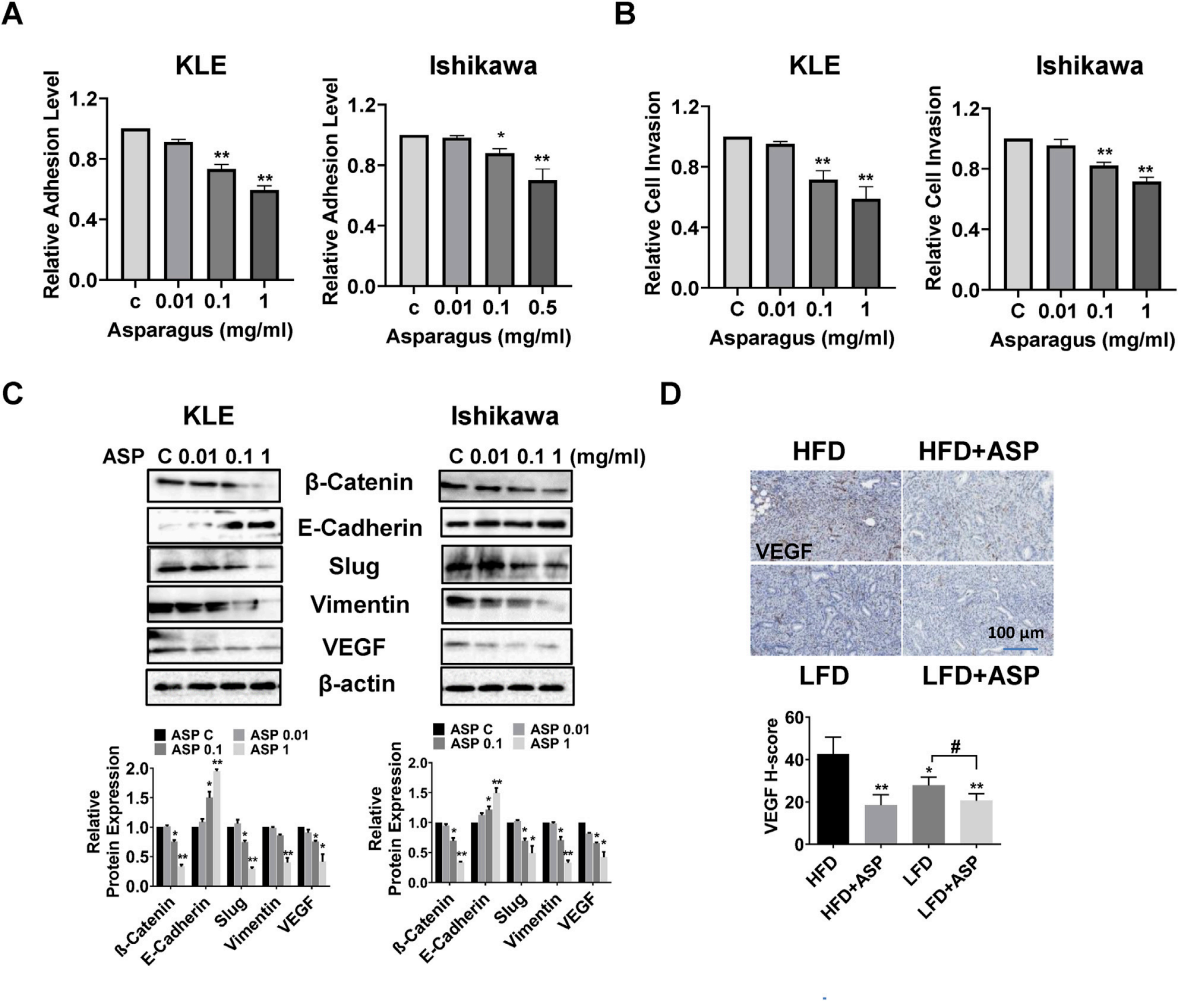
ASP inhibited adhesion and invasion in EC cells and reduced VEGF expression in *LKB1* ^*fl/fl*^ *p53* ^*fl/fl*^ mice ASP extract at doses of 0.1 and 1 mg/mL inhibited cell adhesion in the KLE and Ishikawa cells **(A)**. The transwell assay showed that ASP significantly reduced invasive ability in both cell lines after 6 h of treatment **(B)**. Western blotting assay was used to determine the change of EMT related proteins. ASP increased E-Cadherin expression and decreased the expression of β-Catenin, Vimentin, Slug, and VEGF in both cells **(C)**. IHC staining results showed that ASP reduced the expression of VEGF in EC tissues of obese and lean *LKB1* ^*fl/fl*^ *p53* ^*fl/fl*^ mice **(D)**. *p < 0.05, **p < 0.01, ^#^ *p* < 0.05. * and ** represent comparison with control.

Our previous study found that ASP extract effectively inhibited cell adhesion and invasion and reduced VEGF expression in ovarian cancer cell lines and in a transgenic mouse model of ovarian cancer (Xu et al., 2021). To investigate the role of ASP in cellular adhesion and invasion in EC cells, the KLE and Ishikawa cells were cultured in laminin-1-coated 96-well plates and treated with 0.01, 0.1, and 1 mg/mL ASP extracts for 2 hours. The results showed that ASP significantly inhibited cell adhesion ability in a concentration-dependent manner in both cell lines (Figure 5A). Similarly, ASP extract decreased the invasive ability of the KLE and Ishikawa cells using a transwell migration assay. Cell invasion was reduced by 41.5% and 28.3% in the KLE and Ishikawa cells, respectively, after treatment with 1 mg/mL ASP for 6 h (Figure 5B). To evaluate the effect of ASP on epithelial-to-mesenchymal transition (EMT) and angiogenesis in EC cells, both cell lines were treated with 0.01, 0.1, and 1 mg/mL ASP extracts for 18 h. Western blotting results demonstrated that ASP decreased the expression of VEGF, β-catenin, Slug and vimentin and increased the expression of E-Cadherin in the KLE and Ishikawa cells (Figure 5C). More importantly, in the EC tissues of *Lkb1* ^*fl/fl*^ *p53* ^*fl/fl*^ mice, IHC staining results showed that HFD significantly increased the expression of VEGF compared with LFD mice, and ASP extract effectively reduced VEGF expression in obese and lean conditions (Figure 5D). These results confirm that ASP may reduce adhesion and invasion in EC cells and *Lkb1* ^*fl/fl*^ *p53* ^*fl/fl*^ mice.

### ASP inhibited AKT/mTOR and MAPK pathways in EC cells and *Lkb1* ^*fl/fl*^ *p53* ^*fl/fl*^ mice

**FIGURE 6:**
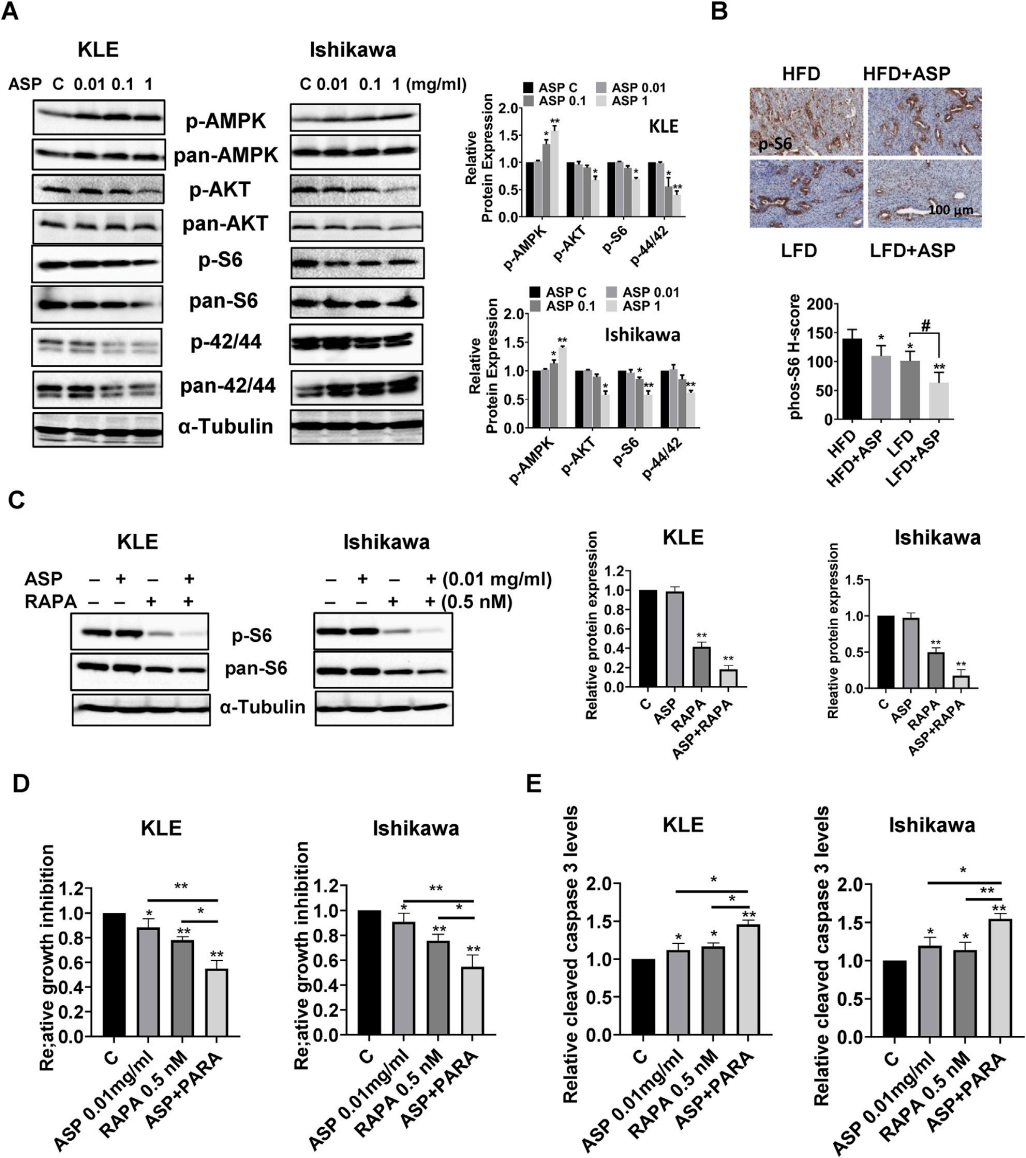
ASP inhibited AKT/mTOR and MAPK pathway in EC. Western blotting results demonstrated that ASP increased phosphorylated AMPK expression and decreased the expression of phosphorylated AKT, p42/44, and S6 in the KLE and Ishikawa cells after 18 h of treatment **(A)**. IHC staining results showed that ASP reduced the expression of phosphorylated S6 in the EC tissues of obese and lean *LKB1* ^*fl/fl*^ *p53* ^*fl/fl*^ mice **(B)**. After both cells were treated with ASP, rapamycin and the combination for 24 h, Western blotting showed that the combination treatment inhibited the expression of phosphorylation of S6 in both cells **(C)**. ASP combined with rapamycin had a stronger inhibitory effect on cell proliferation **(D)** and more effectively increased the level of cleaved caspase 3 in both cells **(E)**. *p < 0.05, **P< 0.01. * and ** represent comparison with control.

Given that AKT/mTOR and MAPK pathways are involved in the anti-tumorigenic activity of ASP in cancer (Zhang et al., 2023; Li et al., 2023; Liang et al., 2022), the effect of ASP on the AKT/mTOR and MAPK pathways in EC cells was detected by Western blotting using phosphorylated AMPK, AKT, p42/44, and S6 antibodies. As shown in Figure 6A, ASP extracts decreased the expression of phosphorylated AKT, p42/44, and S6 and increased the expression of AMPK after the KLE and Ishikawa cells were treated with ASP extracts for 18 h. IHC results demonstrated that HFD significantly promoted the expression of phosphorylated S6 compared with the LFD group. ASP effectively reduced the expression of phosphorylated-S6 in obese and lean mice after 4 weeks of treatment (Figure 6B).

To further investigate the regulatory role of AKT/mTOR/S6 pathway on ASP-induced cell proliferation, the KLE and Ishikawa cells were treated with rapamycin (a mTOR inhibitor), ASP extract, and the combination of ASP and rapamycin overnight. The results of Western blotting confirmed that rapamycin (0.5 nM) significantly reduced phosphorylated S6 expression compared to untreated cells, and the combination of ASP (0.01 mg/mL) and rapamycin showed a more potent inhibitory effect on the expression of phosphorylation of S6 in both cells (Figure 6C). When compared to ASP or rapamycin treatment alone, the combined treatment in both cell lines showed more pronounced inhibitory effects on cell proliferation after 72 h of treatment (Figure 6D). Similar results were found for the effect of combination treatment on cleaved caspase 3 activity in both cells, where ASP combined rapamycin increased more activity of cleaved caspase 3 in both cells compared to single agent after 12 h of treatment (Figure 6E). Overall, these results suggest that the ability of ASP to inhibit cell proliferation is dependent on the PTEN/AKT/S6 pathway in EC.

### ASP combined with cisplatin synergistically inhibited the cell proliferation

**FIGURE 7:**
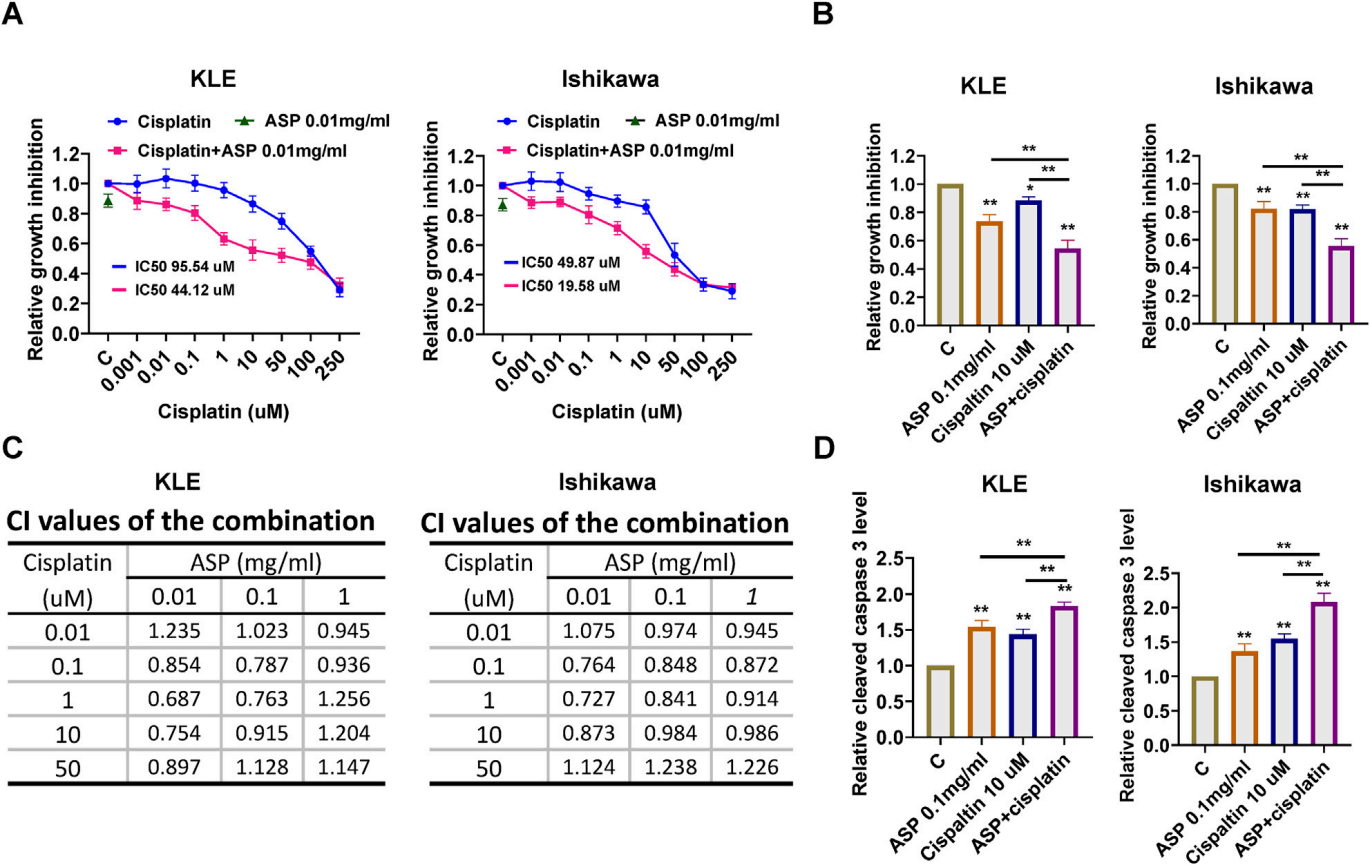
ASP and cisplatin have synergistic effects in inhibiting cell proliferation in EC. The LKE and Ishikawa cells were each treated with ASP (0.01, 0.1, and 1 mg/mL), cisplatin (0.01, 0.1 1, 10 and 50 µM), and a combination for 72 h. MTT assay showed that treatment of cells with ASP (0.01 mg/mL) effectively increased the sensitivity of cells to cisplatin (0.01, 0.1, 1, 10 and 50 µM) **(A)**. The combination of ASP (0.1 mg/mL) and 10 µM cisplatin resulted in greater cell growth inhibition than carboplatin or ascorbate alone in both cells after 72 h of treatment **(B)**. The Bliss independence model was used to calculate the combination index (CI) for each combination group in both cells **(C)**. The combination of ASP (0.1 mg/mL) and cisplatin (10 µM) demonstrated a higher cleaved caspase 3 level than each drug alone after 12 h of treatment in both cells **(D)**. *p < 0.05, **p < 0.01. * and ** represent comparison with control.

Platinum-based chemotherapy is the standard therapeutic strategy for patients with advanced EC. To evaluate the synergistic effect of ASP on cisplatin in EC cells, the KLE and Ishikawa cells were treated with varying concentrations of cisplatin (0.001–250 µM) and then in combination with 0.01, 0.1 and 1 mg/mL ASP for 72 h. MTT assay was used to evaluate cell viability. Relative cell proliferation was reduced in a dose dependent manner following cisplatin treatment. Treatment of both cell lines with 0.01, 0.1 and 1 mg/mL ASP in combination with cisplatin (0.01–50 uM) significantly enhanced the inhibitory effect on cell proliferation compared with ASP or cisplatin alone (Figures 7A, B). The combination index (CI) calculated by CompuSyn software also confirmed that the combination of low-dose ASP and low-dose cisplatin produced a synergistic cytotoxic effect (CI < 1) in the 2 cell lines (Figure 7C). To investigate the effect of the combination of ASP and cisplatin on cleaved caspase 3 activity, ELISA assay showed that the combination of 0.1 mg/mL ASP and 10 uM cisplatin elevated cleaved caspase 3 activity to 1.83-fold compared with 1.55-fold and 1.41 of ASP and cisplatin, respectively, in the KLE cells, and to 2.16-fold compared with 1.37-fold and 1.48-fold of ASP and cisplatin in the Ishikawa cells, respectively (Figure 7D). These results indicate that the combination of ASP and cisplatin produces a synergistic effect in inhibiting cell proliferation in EC cells.

### ASP reduced glycolytic activity in EC cells

**FIGURE 8:**
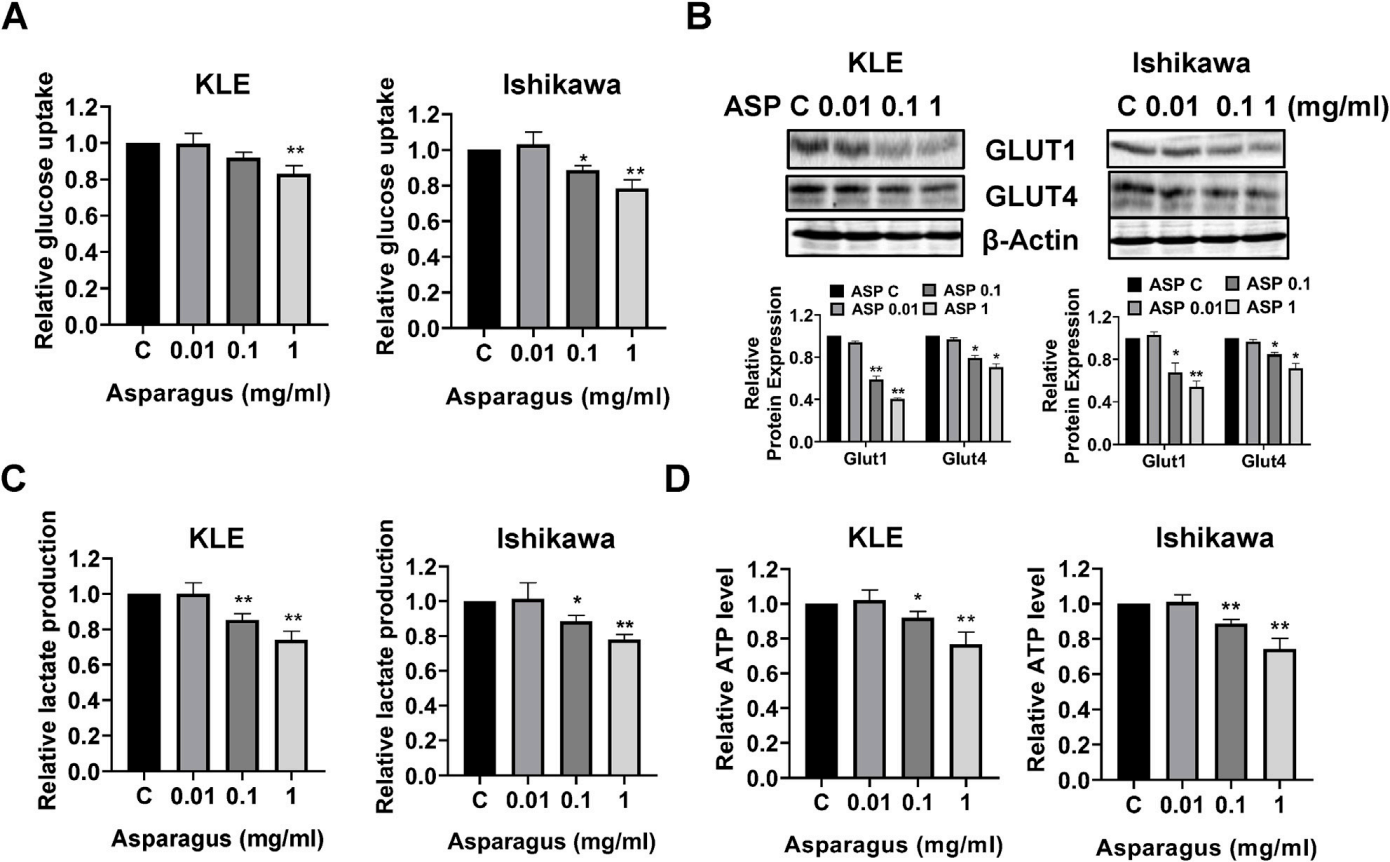
ASP inhibited glycolytic activity in EC cells. After the KLE and Ishikawa cells were treated with 0.01, 0.1, and 1 mg/mL for 12 h, 2-NBDG assay showed that ASP significantly reduced glucose uptake in both cell lines **(A)**. Western blotting results found that ASP extracts decreased the expression of GLUT1 and GLUT4 after treatment for 12 h in both cell lines **(B)**. Cell culture media of KLE and Ishikawa cells were used to measure the production of lactate. ASP effectively reduced the production of lactate in both cells after 12 h of treatment **(C)**. ASP decreased cellular ATP level in the KLE and Ishikawa cells after 24 h of treatment **(D)**. *p < 0.05, **P< 0.01. * and ** represent comparison with control.

Given the hypoglycemic effect of ASP in diabetic rats (Zhao et al., 2011; Hafizur et al., 2012), the effect of ASP extract on glycolysis was investigated in the KLE and Ishikawa cells. 1 mg/mL ASP significantly reduced glucose uptake by nearly 17.8% in the KLE cells and by 22.1% in the Ishikawa cells after 12 h of treatment compared with control cells (Figure 8A). Western blotting results showed that ASP decreased the expression of GLUT 1 and GLUT 4 in both cell lines after 12 h of treatment (Figure 8B). Finally, we measured the production of lactate in the culture media and the cellular ATP levels by ELISA assays. ASP decreased lactate production by 21.2% in the KLE cells and 26.4% in the Ishikawa cells in cell culture media compared to untreated cells, respectively (Figure 8C). In addition, 1 mg/mL ASP significantly reduced cellular ATP level by 23.3% in the KLE and 25.6% in the Ishikawa cells, respectively, after 24 h of treatment (Figure 8D, *p* < 0.01). These results support the ability of ASP to reduce glycolytic activity in EC cells.’

## Discussion

The metabolites of natural products have been widely reported for their anti-tumorigenic potential in multiple types of cancer in pre-clinical models and some clinical trials (Sharifi-Rad et al., 2019). The main bioactive metabolites of the ASP extract related to its beneficial biological activities include polysaccharides, steroidal saponins, and flavonoids, which exert their anti-tumorigenic effects through a complex range of molecular targets and cell signaling pathways in cancer cells and animal models. Different parts of ASP appear to have different concentrations and ratios of metabolites and may exhibit different biological functions (Lee et al., 2014; Witaszak et al., 2020). Our ASP test reports showed that ASP extract contained high levels of saponins, rutin, quercetin, amaranth, resveratrol, phenolic compounds, polysaccharides, and free amino acids (Table 1). ASP extract significantly induced cell cycle G1 arrest in breast MCF-7 cells without apoptosis induction but did not affect MDA-MB231 cell cycle changes (Romani et al., 2021). Asparanin A isolated from ASP caused cell cycle G1 arrest in Ishikawa cells, induced G2/M cell cycle arrest in human hepatocellular carcinoma HepG2 cells, and induced apoptosis in a dose-dependent manner in both cells (Liu et al., 2009; Zhang et al., 2020). A previous study from our group has shown that ASP extract inhibited cell proliferation through the induction of cell cycle G1 arrest and mitochondrial apoptosis in a dose-dependent manner in ovarian cancer cells (Xu et al., 2021). Similarly, in the present study, we found that ASP extract induced G1 phase arrest, increased Annexin V expression and activities of caspase 3, 8, and 9, decreased the expression of Bcl-2 and Mcl-1, and inhibited AKT/mTOR and MAPK pathways. Cell cycle arrest is thought to be an active response instigated by noxious stress stimuli or DNA damage to facilitate DNA repair, and the interplay of the CDK family and the Bcl-2 family has putative roles in the regulation of apoptosis in unique ways in cancer cells (Terrano et al., 2010; Zinkel et al., 2006). Bcl-2 not only regulate intrinsic apoptotic pathways but also participate in cell cycle progression through multiple mechanisms, including interacting CDKs and E2F (Hatok and Racay, 2016; Hardwick and Soane, 2013). Our study supports that cell cycle G1 arrest and apoptosis are involved in ASP-induced cell growth inhibition, and these processes may be dependent on the AKT/mTOR and MAPK pathways.

Cellular redox status has been shown to have key cellular functions in regulating cellular metabolism, proliferation, differentiation, transcription, and apoptosis, and the delicate balance between oxidized and reduced states is critical for the proper function and survival of cells (Le Gal et al., 2021; Menon and Goswami, 2007). ASP appears to have dual functions as a pro-oxidant and an antioxidant in normal and cancer cells. For example, ASP extract decreased ROS generation and DNA damage and increased glutathione (GSH) synthesis through the improvement of HSP70-mediated redox balance in bovine cumulus-granulosa cells (Ho et al., 2021). Treatment of mouse skin fibroblast L929 cells and human hepatoma HepG2 cells with ASP extract significantly enhanced the protection against H_2_O_2_-induced oxidative damage and H_2_O_2_-induced MMP-9 mRNA expression, respectively (Shirato et al., 2016; Zhang et al., 2019). However, our recent studies found that treatment of ovarian cancer cell lines with ASP extract significantly increased intracellular ROS production and reduced mitochondrial membrane potential in a dose-dependent manner, suggesting a function of ASP to induce cellular stress in ovarian cancer cells (Xu et al., 2021; Zhang et al., 2023). Our current results also confirm that ASP extract enhanced cellular ROS levels and decreased TMRE productions, accompanied by an increase in the expression of PERK and Bip in EC cells. Treatment of obese and lean *Lkb1* ^*fl/fl*^ *p53* ^*fl/fl*^ mice with ASP extract effectively increased the expression of Bip in EC tissues. Pretreated EC cells with NAC significantly revised ASP-induced cell stress, cleaved caspase 3 and inhibition of cell proliferation. The ASP-induced oxidative stress observed in this study further supports the role of ASP extract in inhibiting cell proliferation and tumor growth, as oxidative stress leads to cell cycle arrest in the G1 or G2 phase and triggers apoptosis in cancer cells (Lee et al., 2019; Matés et al., 2012).

Our previous studies found that ASP significantly reduced the cellular adhesive and invasive abilities of ovarian cancer cells and decreased the expression of VEGF in tumor tissues of a transgenic mouse model of ovarian cancer (Xu et al., 2021; Zhang et al., 2023). Similarly, the present results demonstrate that ASP inhibited cell adhesion and invasion, accompanied by inhibition of EMT processes in EC cells, and reduced the expression of VEGF in endometrial tumor tissues in *Lkb1* ^*fl/fl*^ *p53* ^*fl/fl*^ mice. Inhibition of MMP9 expression in colorectal cancer cells and reduction of cell migration and invasion in breast cancer cells have been documented following treatment with ASP extracts (Romani et al., 2021; Wang et al., 2013; Paganelli et al., 2021). Cell invasion during cancer progression has been shown to be critically dependent on the acquisition of the EMT process, and enhanced enzymatic degradation of extracellular matrix components by MMPs induces cell invasion and tumor progression; thus, interfering with cell invasion, the EMT process, and reduction in the expression of MMPs is essential for reducing tumor metastases (Son and Moon, 2010; Barillari, 2020). Several studies have demonstrated the importance of ASP extracts in anti-invasiveness, regulating the EMT process, and reducing angiogenesis in preclinical models of cancer (Romani et al., 2021; Paganelli et al., 2021). Our results and those of other studies suggest that ASP may inhibit tumor metastasis, which is worthy of further study in animal cancer models.

ASP is a diabetes-friendly vegetable with a long history as a folk remedy for hyperglycemia and diabetes (Hafizur et al., 2012). Treatment of streptozotocin (STZ)-induced diabetic rats with ASP extracts for 21 days significantly reduced fasting serum glucose and glucose tolerance and increased the hepatic glycogen level (Zhao et al., 2011). Furthermore, Hafizur et al. reported that ASP extract exhibited antidiabetic effects by improving insulin secretion and β-cell function as well as antioxidant status, similar to those of glibenclamide in STZ-induced diabetic rats (Hafizur et al., 2012). A recent study showed that ASP extract effectively reduced the activities of G6PD, LDHA, PKM2, c-Myc, and glutaminase in HepG2 cells, suggesting that ASP may inhibit glycolysis and glutamine metabolism in cancer cells (Al-Shafie et al., 2023). Given that obesity and diabetes are important risk factors for EC and high glucose conditions promote EC cell proliferation (King et al., 2023; Han et al., 2015), we investigated the effect of ASP on glycolysis in EC cells. Our results showed that ASP reduced glucose uptake and lactate production in EC cells, while decreasing the expression of PKM2 and LDHA and cellular ATP levels, confirming that ASP extract can inhibit glycolysis in EC cells. It is well known that some metabolites in natural products, such as polysaccharides, flavonoids, saponins, terpenes, glycosides, and sterols, have hypoglycemic activity (Li et al., 2004; Mukherjee et al., 2006; Wang and Ng, 1999). Thus, the inhibition of glycolysis may be another anti-tumor mechanism of ASP.

Given that plant-based natural products have significant tolerability and fewer adverse side effects, the combination of natural products with conventional chemotherapy is an ideal treatment approach for the prevention and treatment of cancer (Chaudhry et al., 2022). ASP in combination with extract of Lentinula edodes mycelia was synergistic in inhibiting cell growth, inducing apoptosis, and reducing invasion in colorectal cancer cells (Paganelli et al., 2021). Deproteinized asparagus polysaccharides sensitized the antitumor effects of mitomycin in hepatocellular carcinoma cells and mouse xenograft models of hepatocellular carcinoma (Xiang et al., 2014), In ovarian cancer, ASP combined with paclitaxel synergistically inhibited cell proliferation, induced cellular stress and apoptosis, and reduced cell invasion through DNA damage pathways and suppressing microtubule dynamics in paclitaxel-sensitive and -resistant ovarian cancer cell lines (Xu et al., 2021; Zhang et al., 2023). The present results indicate that the combination of ASP and cisplatin at low doses produced a significant synergistic effect on cell growth and increased the production of cleaved caspase 3 compared with ASP or cisplatin alone in both cells. Although the molecular mechanism of the enhanced synergistic effect of ASP and cisplatin is still unclear, it is worthwhile to explore this synergistic effect using ASP extract in animal studies.

## Conclusion

Although ASP is widely used and valued as a healthy vegetable, the beneficial roles of its various metabolites in certain diseases, especially cancer, have only recently received special attention. Our preliminary results showed that ASP extract exhibited anti-proliferative activity, reduced glycolytic activity, and synergistically increased sensitivity to cisplatin in EC cells. Importantly, ASP extract significantly inhibited tumor growth in both obese and lean *LKB1* ^*fl/fl*^ *p53* ^*fl/fl*^ mice, with obese mice responding better to ASP treatment. Although we only used two EC cell lines and a transgenic mouse model of EC to study the anti-tumor activity of ASP and did not explore the effect of ASP on cell growth in primary cultures of EC, we believe that our findings provide solid preclinical evidence for the use of ASP extract as a potential therapeutic agent to improve chemotherapy in clinical trials for EC patients.

## Data Availability

The original contributions presented in the study are included in the article/Supplementary Material, further inquiries can be directed to the corresponding authors.

## Abbreviations

EC, endometrial cancer; AMPK, AMP-activated protein kinase,; VEGF, vascular endothelial growth factor; ASP, asparagus officinalis; MTT, microculture Tetrazolium.; TMRE, tetramethylrhodamine, ethyl ester.; 2-NBDG, 2-N-(7-Nitrobenz-2-oxa-1,3-diazol-4-yl) Amino)-2-Deoxyglucose; SD-PAGE, sodium dodecyl sulfate–polyacrylamide gel electrophoresis.; HFD, high-fat diet; LFD, low-fat diet; PI, propidium Iodide; IHC, immunohistochemistry; MAPK, mitogen-activated protein kinase.; EMT, epithelial-mesenchymal transition.; FBS, fetal bovine serum.; NAC, N-acetylcysteine.
