# Supplementary material for: Asparagus officinalis L. extract exhibits anti-proliferative and anti-invasive effects in endometrial cancer cells and a transgenic mouse model of endometrial cancer
Source: Front Pharmacol. 2024 Dec 4;15:1507042. doi: 10.3389/fphar.2024.1507042 (PMC11653357; doi:10.3389/fphar.2024.1507042)
Supplement: Supplementary file 1 [file DataSheet2.pdf]

Figure 2

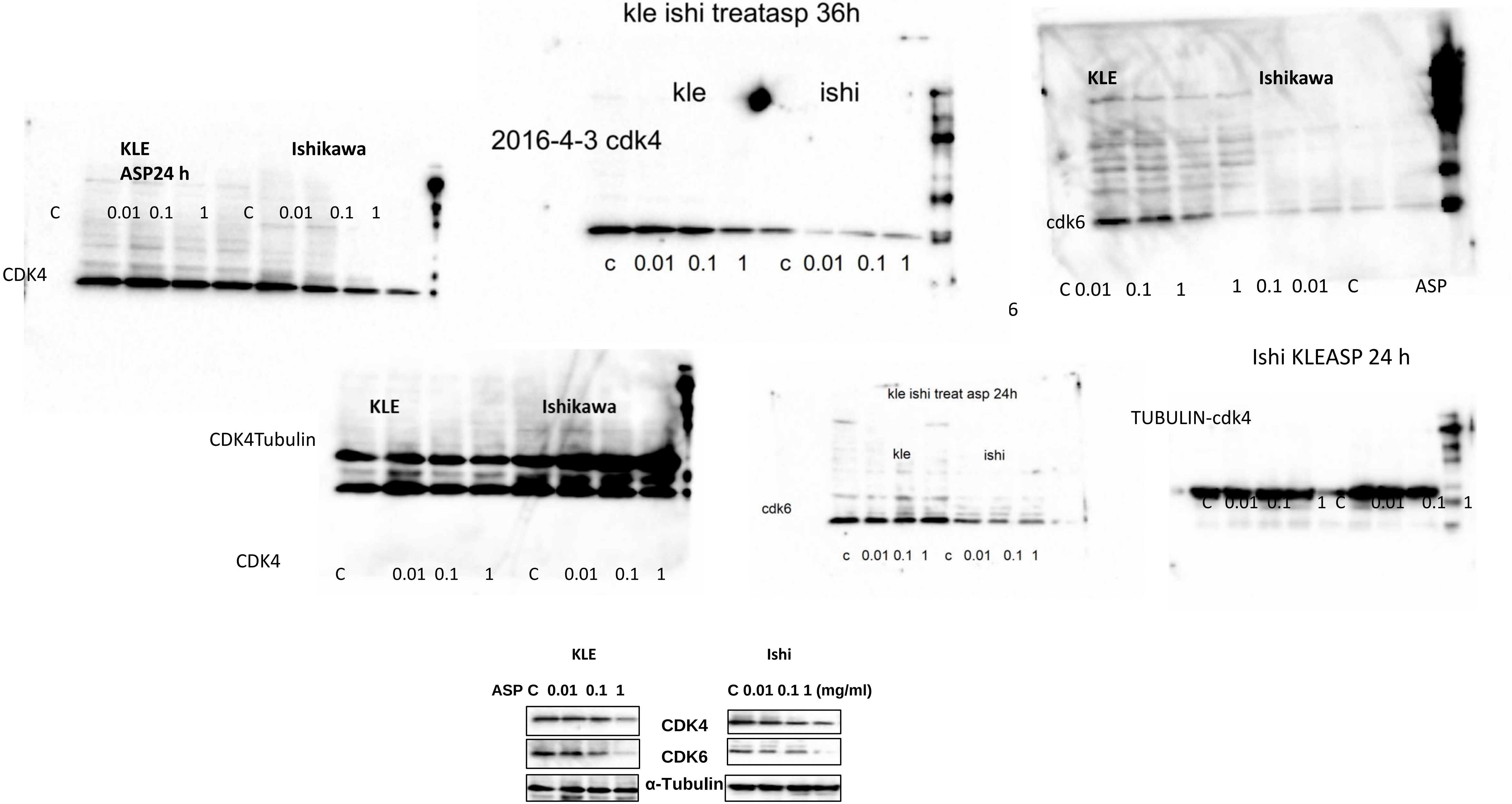

kle ishi treatasp 36h
kle
ishi
2016-4-3 cdk4
c 0.01 0.1 1 c 0.01 0.1 1
KLE
ASP24 h
Ishikawa
C 0.01 0.1 1 C 0.01 0.1 1
CDK4
KLE
Ishikawa
cdk6
C 0.01 0.1 1 1 0.1 0.01 C ASP
6
Ishi KLEASP 24 h
KLE
Ishikawa
CDK4Tubulin
CDK4
C 0.01 0.1 1 C 0.01 0.1 1
kle ishi treat asp 24h
kle
ishi
cdk6
c 0.01 0.1 1 c 0.01 0.1 1
TUBULIN-cdk4
C 0.01 0.1 1 C 0.01 0.1 1
KLE
Ishi
ASP C 0.01 0.1 1
C 0.01 0.1 1 (mg/ml)
CDK4
CDK6
α-Tubulin

Figure 3

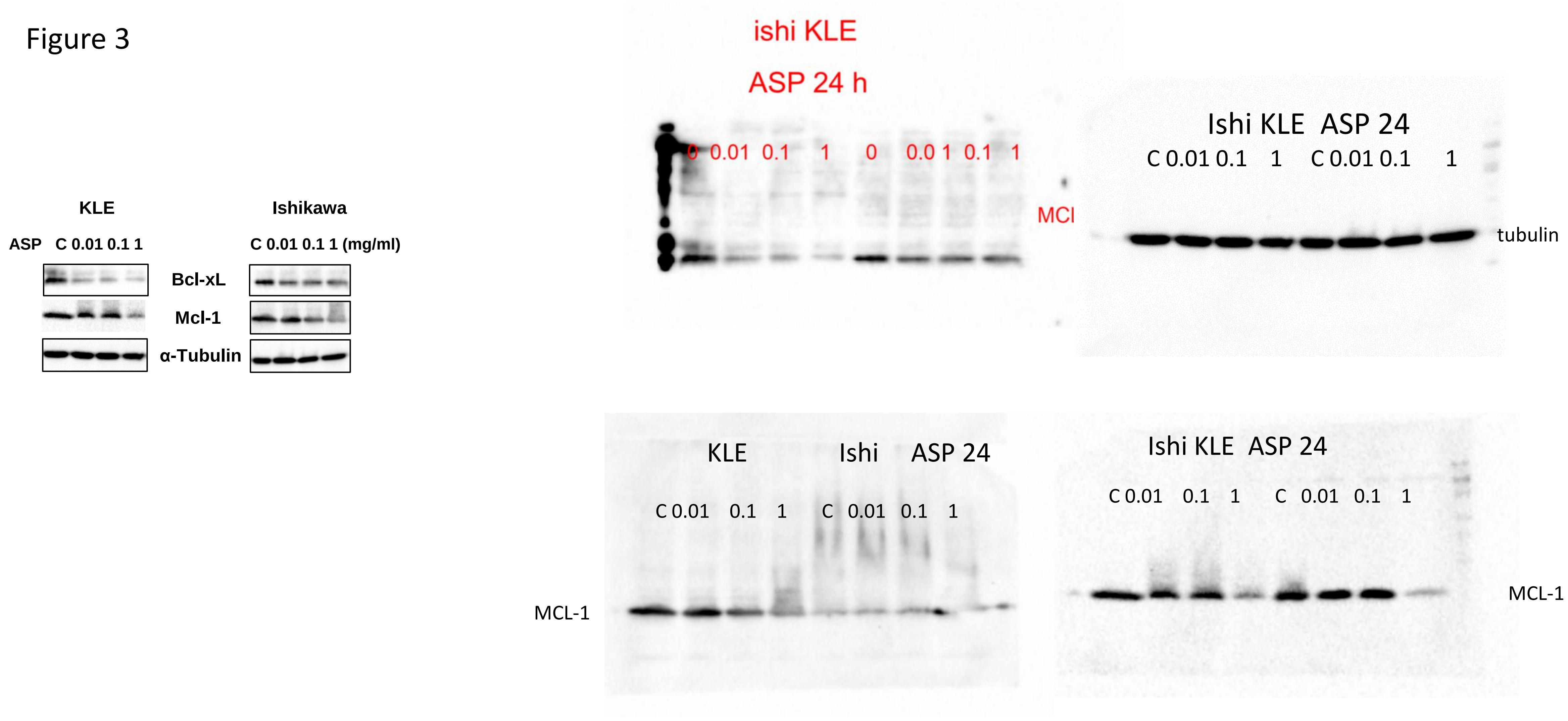
KLE
Ishikawa
ASP C 0.01 0.1 1
C 0.01 0.1 1 (mg/ml)
Bcl-xL
Mcl-1
α-Tubulin
ishi KLE
ASP 24 h
0 0.01 0.1 1 0 0.01 0.1 1
MCl
Ishi KLE ASP 24
C 0.01 0.1 1 C 0.01 0.1 1
tubulin
KLE Ishi ASP 24
C 0.01 0.1 1 C 0.01 0.1 1
MCL-1
Ishi KLE ASP 24
C 0.01 0.1 1 C 0.01 0.1 1
MCL-1

Figure 4

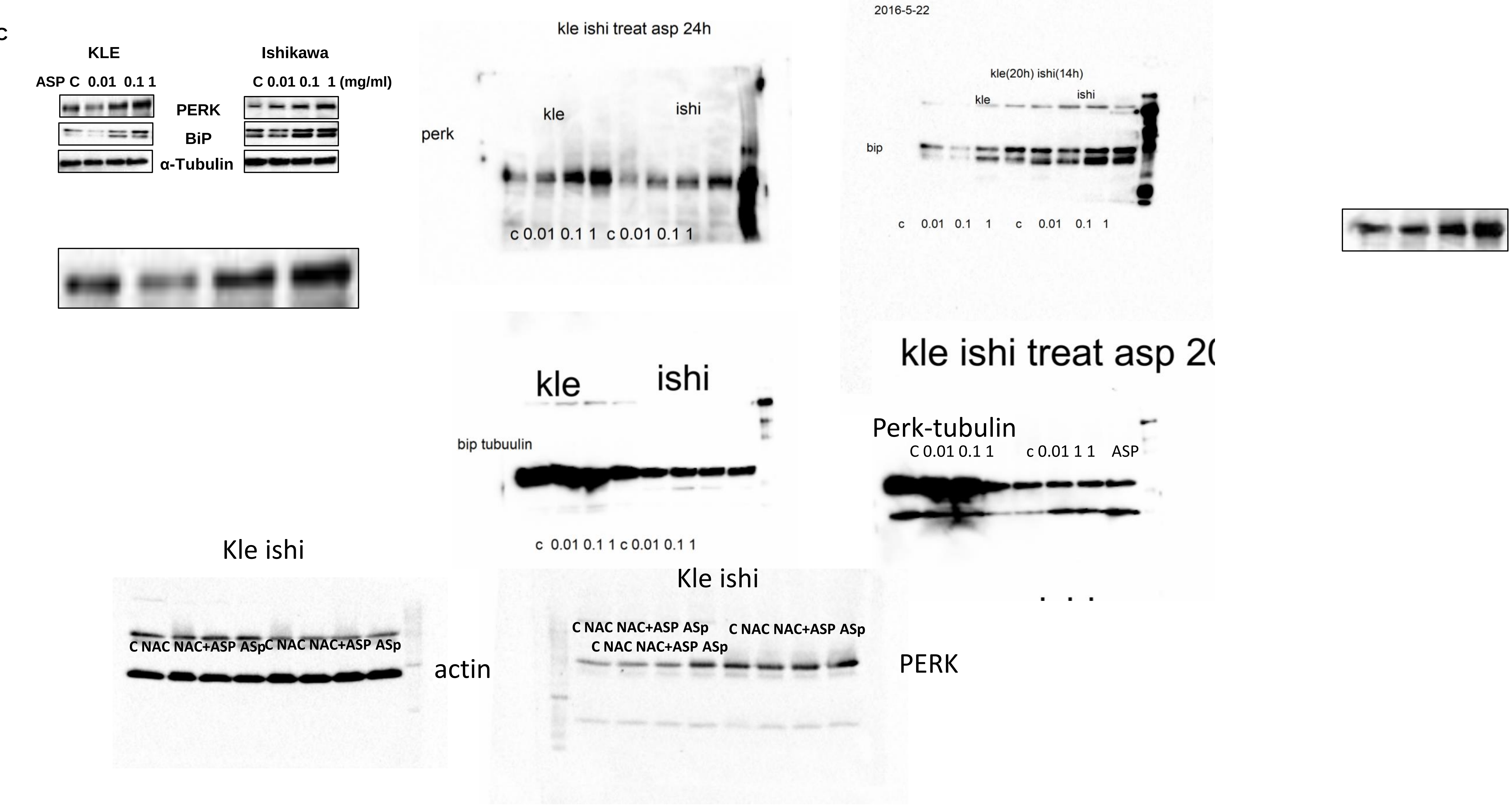
C
KLE
Ishikawa
ASP C 0.01 0.1 1
C 0.01 0.1 1 (mg/ml)
PERK
BiP
α-Tubulin
kle ishi treat asp 24h
perk
kle
ishi
c 0.01 0.1 1 c 0.01 0.1 1
2016-5-22
kle(20h) ishi(14h)
kle
ishi
bip
c 0.01 0.1 1 c 0.01 0.1 1
kle ishi treat asp 2(
kle
ishi
bip tubuulin
c 0.01 0.1 1 c 0.01 0.1 1
Perk-tubulin
C 0.01 0.1 1 c 0.01 1 1 ASP
Kle ishi
C NAC NAC+ASP ASp C NAC NAC+ASP ASp
actin
Kle ishi
C NAC NAC+ASP ASp C NAC NAC+ASP ASp
C NAC NAC+ASP ASp
PERK

Figure 5

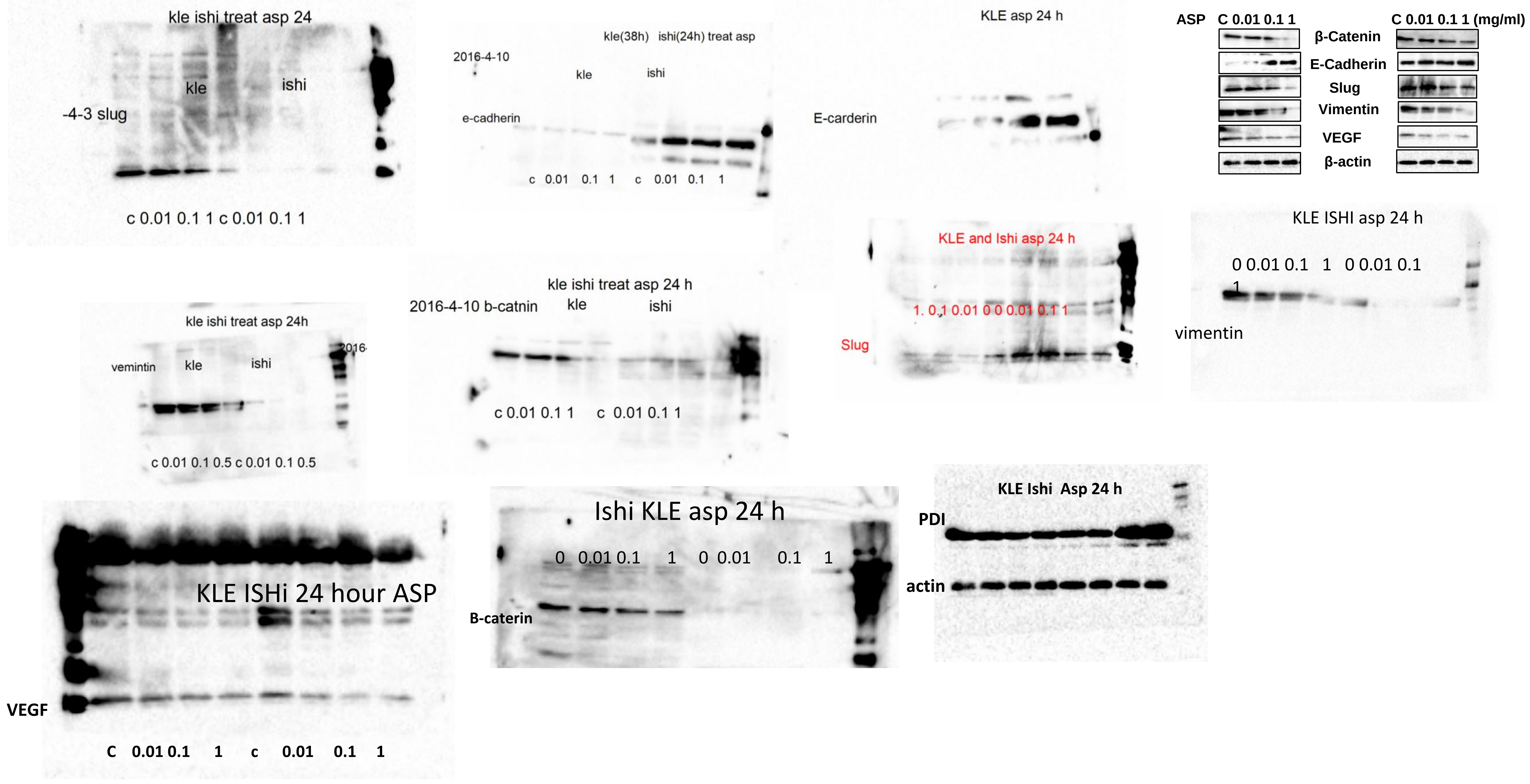

kle ishi treat asp 24
kle
ishi
-4-3 slug
c 0.01 0.1 1 c 0.01 0.1 1
kle(38h) ishi(24h) treat asp
2016-4-10
kle
ishi
e-cadherin
c 0.01 0.1 1 c 0.01 0.1 1
KLE asp 24 h
E-carderin
KLE
Ishikawa
ASP
C 0.01 0.1 1
C 0.01 0.1 1 (mg/ml)
β-Catenin
E-Cadherin
Slug
Vimentin
VEGF
β-actin
KLE and Ishi asp 24 h
1. 0.1 0.01 0 0 0.01 0.1 1
Slug
KLE ISHI asp 24 h
0 0.01 0.1 1 0 0.01 0.1 1
vimentin
kle ishi treat asp 24h
vemintin
kle
ishi
c 0.01 0.1 0.5 c 0.01 0.1 0.5
kle ishi treat asp 24 h
2016-4-10 b-catnin
kle
ishi
c 0.01 0.1 1 c 0.01 0.1 1
KLE ISHi 24 hour ASP
VEGF
C 0.01 0.1 1 c 0.01 0.1 1
Ishi KLE asp 24 h
0 0.01 0.1 1 0 0.01 0.1 1
B-caterin
KLE Ishi Asp 24 h
PDI
actin

Figure 6

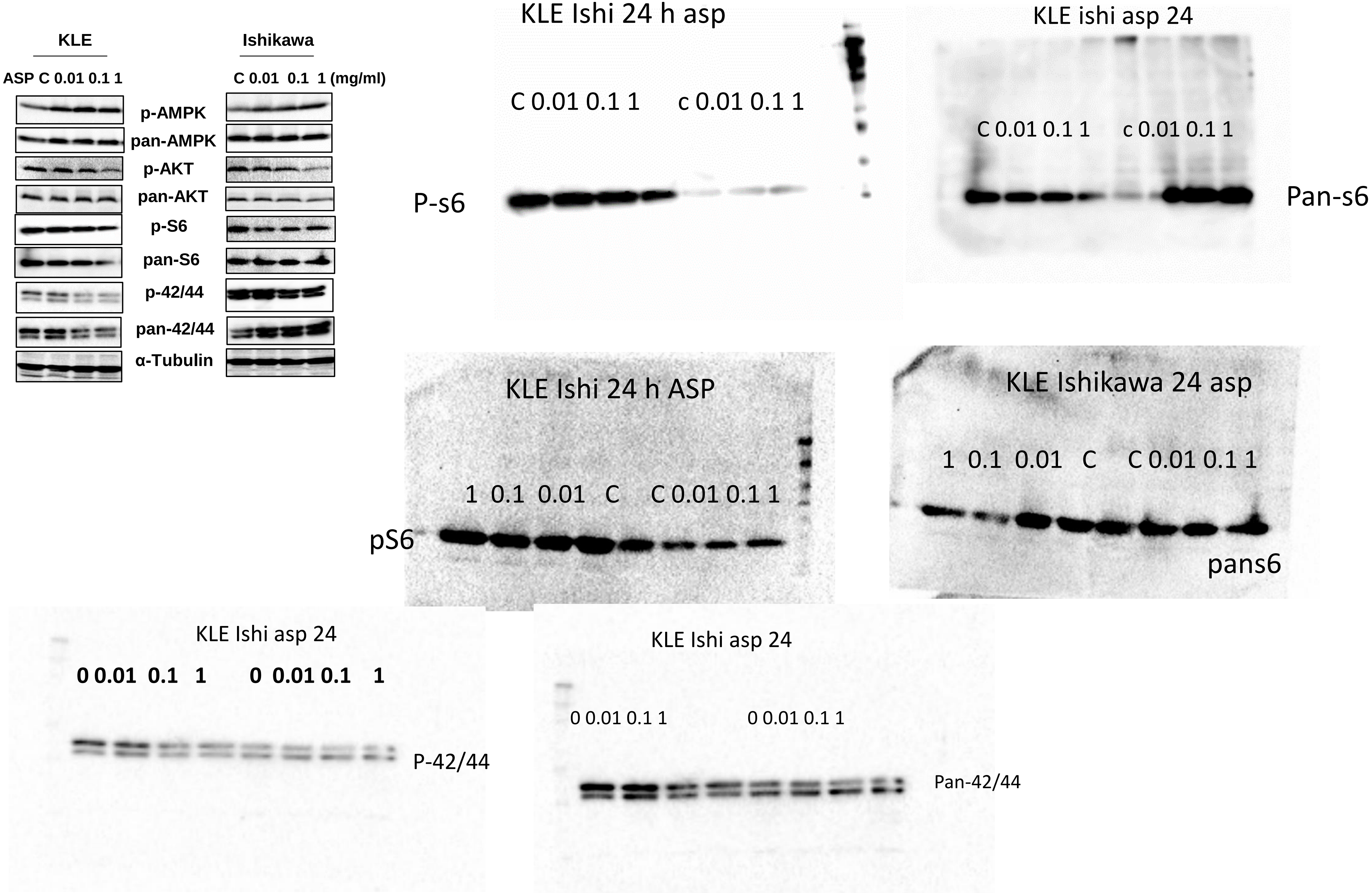
KLE
Ishikawa
ASP C 0.01 0.1 1
C 0.01 0.1 1 (mg/ml)
p-AMPK
pan-AMPK
p-AKT
pan-AKT
p-S6
pan-S6
p-42/44
pan-42/44
α-Tubulin
KLE Ishi 24 h asp
C 0.01 0.1 1 c 0.01 0.1 1
P-s6
KLE ishi asp 24
C 0.01 0.1 1 c 0.01 0.1 1
Pan-s6
KLE Ishi 24 h ASP
1 0.1 0.01 C C 0.01 0.1 1
pS6
KLE Ishikawa 24 asp
1 0.1 0.01 C C 0.01 0.1 1
pans6
KLE Ishi asp 24
0 0.01 0.1 1 0 0.01 0.1 1
P-42/44
KLE Ishi asp 24
0 0.01 0.1 1 0 0.01 0.1 1
Pan-42/44

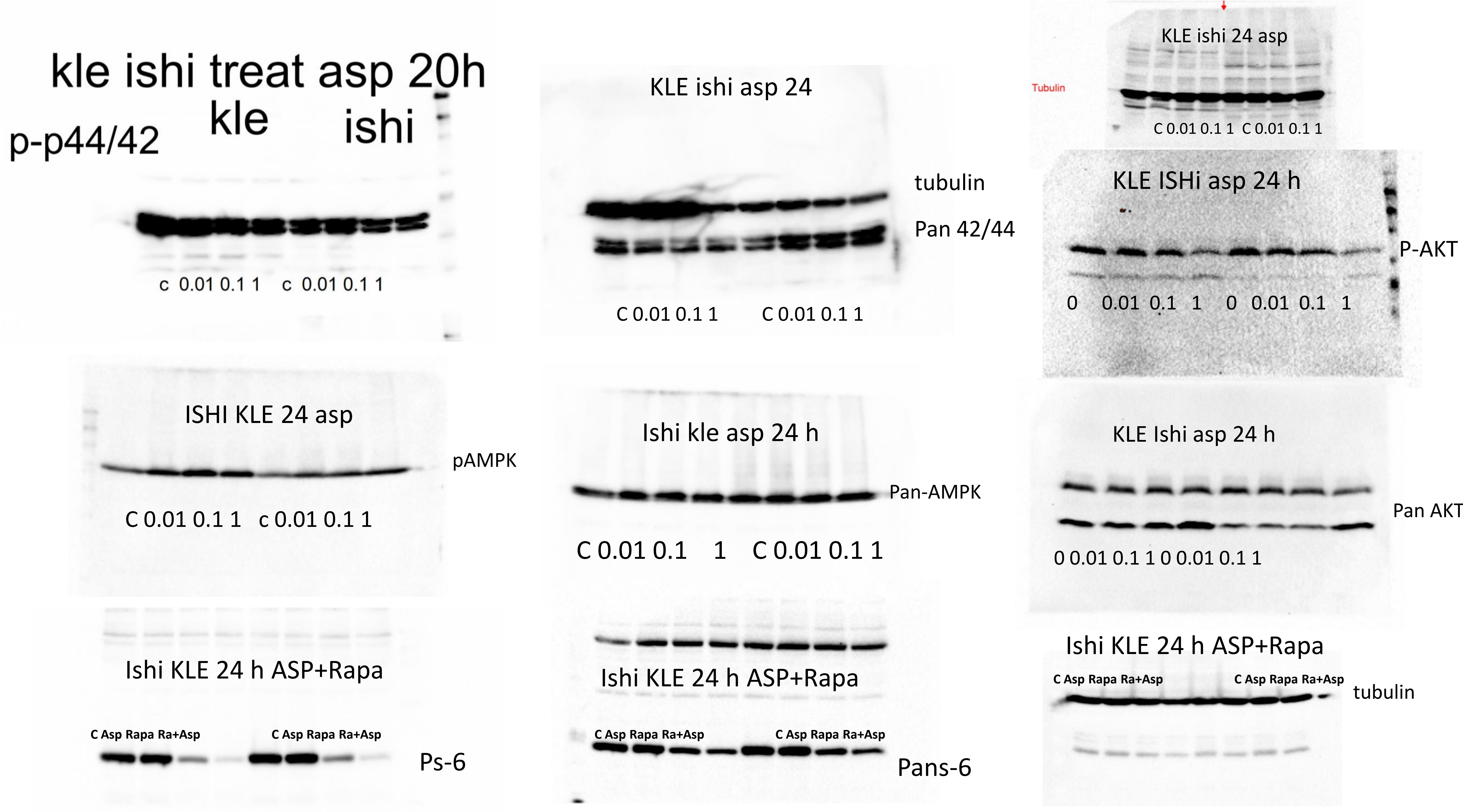
kle ishi treat asp 20h
kle
ishi
p-p44/42
c 0.01 0.1 1 c 0.01 0.1 1
KLE ishi asp 24
tubulin
Pan 42/44
C 0.01 0.1 1 C 0.01 0.1 1
KLE ishi 24 asp
Tubulin
C 0.01 0.1 1 C 0.01 0.1 1
KLE ISHi asp 24 h
P-AKT
0 0.01 0.1 1 0 0.01 0.1 1
ISHI KLE 24 asp
pAMPK
C 0.01 0.1 1 c 0.01 0.1 1
Ishi kle asp 24 h
Pan-AMPK
C 0.01 0.1 1 C 0.01 0.1 1
KLE Ishi asp 24 h
Pan AKT
0 0.01 0.1 1 0 0.01 0.1 1
Ishi KLE 24 h ASP+Rapa
C Asp Rapa Ra+Asp C Asp Rapa Ra+Asp
Ps-6
Ishi KLE 24 h ASP+Rapa
C Asp Rapa Ra+Asp C Asp Rapa Ra+Asp
Pans-6
Ishi KLE 24 h ASP+Rapa
C Asp Rapa Ra+Asp C Asp Rapa Ra+Asp
tubulin

Figure 8

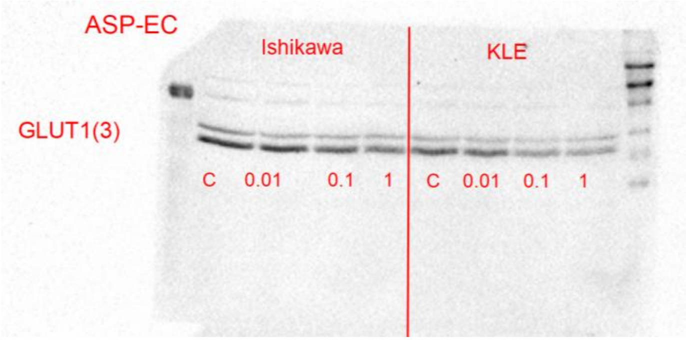
ASP-EC
Ishikawa
KLE
GLUT1(3)
C 0.01 0.1 1 C 0.01 0.1 1

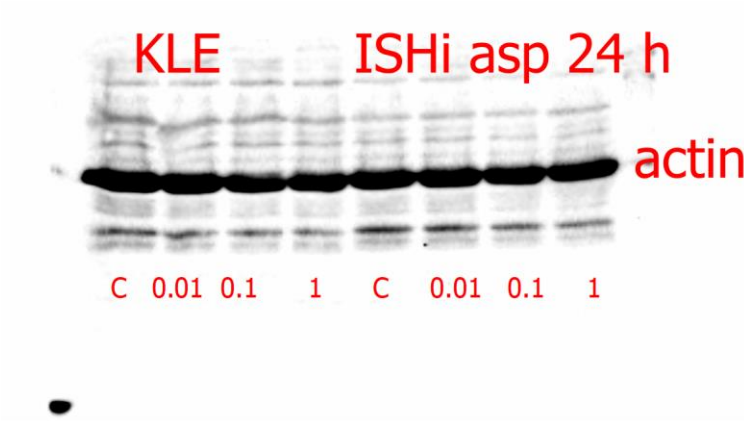
KLE ISHi asp 24 h
actin
C 0.01 0.1 1 C 0.01 0.1 1

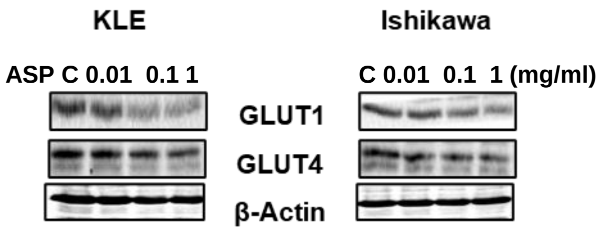
KLE
Ishikawa
ASP C 0.01 0.1 1
C 0.01 0.1 1 (mg/ml)
GLUT1
GLUT4
β-Actin

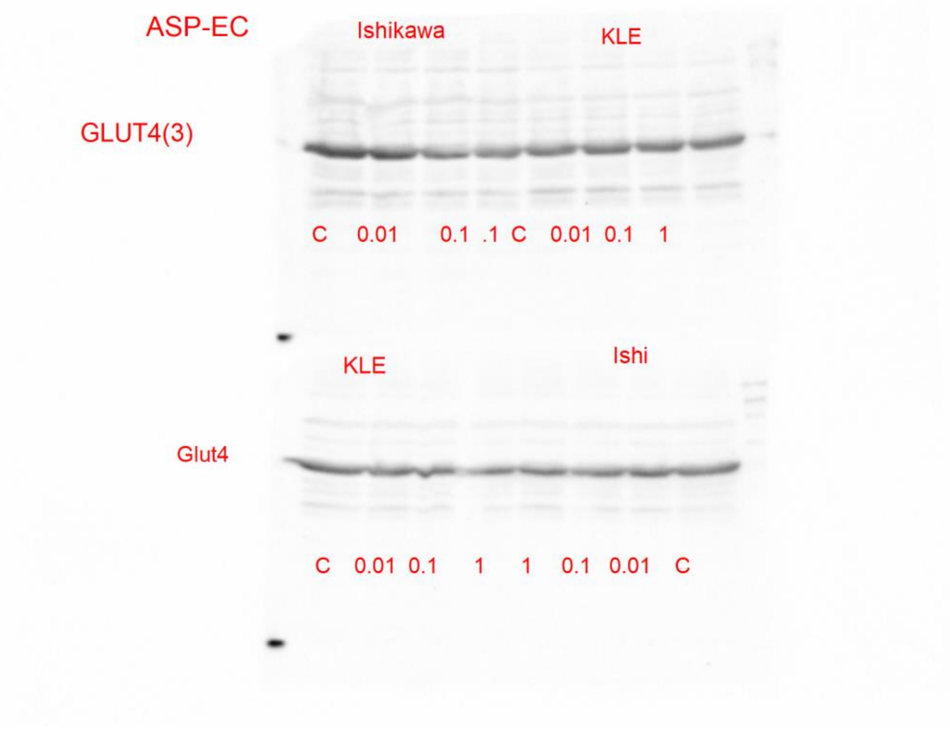
ASP-EC
Ishikawa
KLE
GLUT4(3)
C 0.01 0.1 .1 C 0.01 0.1 1
KLE
Ishi
Glut4
C 0.01 0.1 1 1 0.1 0.01 C
