# Supplementary material for: Asparagus officinalis L. extract exhibits anti-proliferative and anti-invasive effects in endometrial cancer cells and a transgenic mouse model of endometrial cancer
Source: Front Pharmacol. 2024 Dec 4;15:1507042. doi: 10.3389/fphar.2024.1507042 (PMC11653357; doi:10.3389/fphar.2024.1507042)
Supplement: Supplementary file 2 [file Table1.pdf]

Supplemental Table 1. Preparation of the ASP extracts

Information	Description
Species	*Asparagus officinalis* L. Abbreviation: ASP
Research Title	Asparagus officinalis extract exhibits anti-proliferative and anti-invasive effects in vitro and in vivo
Abstract	This study utilized ASP extract to investigate its effects on cell proliferation and tumor growth in endometrial cancer cell lines and a transgenic mouse model of endometrial cancer. ASP extract exhibits meaningful antitumor activity and synergistic effects with cisplatin.
Resource of ASP	ASP was raised and harvested from Juxinyuan Asparagus Industry Development Research Institute, HeZe, Shandong, China.
Harvest Season	Spring harvest.
Harvest location	Chaoxian, Shandong, China. GPS location:35°14′03″N 115°28′47″E.
Authentication of ASP	ASP was identified by technicians in the Juxinyuan Asparagus Industry Development Research Institute
Potential infestation of ASP	ASP was extracted from healthy Asparagus officinalis. No signs of infestation.
Extraction process of ASP	1.Crushed stems and shoots of ASP. 2. Rinsed in distilled water at 95°C. 3. Squeezed the stems and shoots using squeezer equipment. 4. Removed insoluble solids by centrifugation. 5. Concentrated the solution by vacuum to produce 20% soluble solids. 6. The 20% soluble solids solution was further concentrated using a water bath to produce an ASP extract containing 70% soluble components. 7. Sterilized the ASP extracts through pasteurization.
