# Supplementary material for: Asparagus officinalis L. extract exhibits anti-proliferative and anti-invasive effects in endometrial cancer cells and a transgenic mouse model of endometrial cancer
Source: Front Pharmacol. 2024 Dec 4;15:1507042. doi: 10.3389/fphar.2024.1507042 (PMC11653357; doi:10.3389/fphar.2024.1507042)
Supplement: Supplementary file 3 [file Table2.pdf]

Supplemental Table 2. Analytical methods for metabolites in the ASP extracts

Metabolites	Analytical Methods
Rutin	High-performance liquid chromatography (HPLC)
Quercetin	HPLC
sarsagenin	HPLC
phenolic compounds	HPLC
resveratrol	HPLC
Saponins	Spectrophotometry
Free amino acids	HPLC– diode array detector (DAD) method
molybdenum	Inductively coupled plasma mass spectrometry (ICP-MS)
selenium	Inductively coupled plasma mass spectrometry (ICP-MS)
Polysaccharides	Spectrophotometry
