# Supplementary material for: Asparagus officinalis L. extract exhibits anti-proliferative and anti-invasive effects in endometrial cancer cells and a transgenic mouse model of endometrial cancer
Source: Front Pharmacol. 2024 Dec 4;15:1507042. doi: 10.3389/fphar.2024.1507042 (PMC11653357; doi:10.3389/fphar.2024.1507042)
Supplement: Supplementary file 4 [file DataSheet1.pdf]

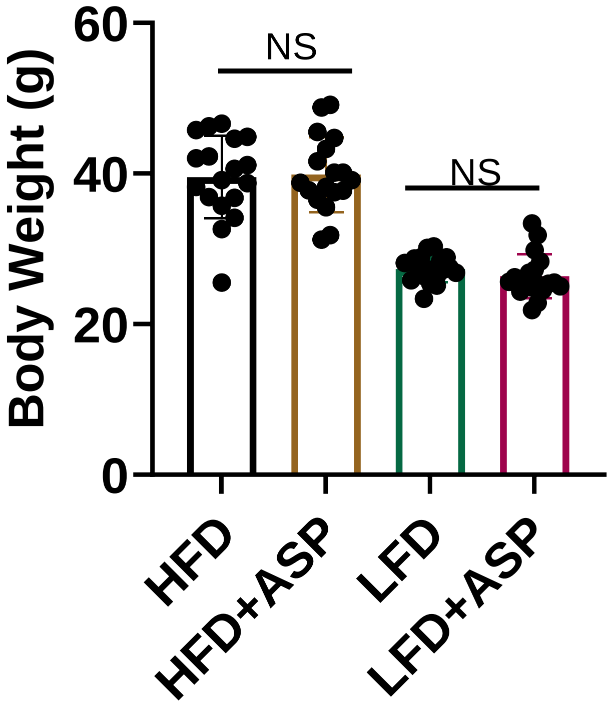

Supplemental Figure 1. Treatment of obese and lean LKB1$^{fl/fl}$ p53 $^{fl/fl}$ mice with ASP extracts (200 mg/kg, oral, daily) for 4 weeks. ASP did not affect body weight changes in obese and lean mice.
